# Inhibition of Abnormal Elevated α‐Synuclein Ameliorates Dopaminergic Neuron Degeneration in Parkinson's Disease Mouse Model

**DOI:** 10.1111/acel.70717

**Published:** 2026-09-23

**Authors:** Xuenan Wang, Yiru Ding, Lujuan He, Xizhi Kang, Xiwen Geng, Fumika Sakaue, Takanori Yokota, Liang‐wen Zhang, Wei Yao, Ji‐chun Zhang

**Affiliations:** ^1^ Institute of Brain Science and Brain‐Inspired Research, Shandong Provincial Key Laboratory of Brain Science Shandong First Medical University & Shandong Academy of Medical Sciences Jinan China; ^2^ Department of Physiology, School of Medicine Jinan University Guangzhou China; ^3^ NHC Key Laboratory of Mental Health (Peking University), and National Clinical Research Center for Mental Disorders (Peking University Sixth Hospital), Peking University Sixth Hospital, Peking University Institute of Mental Health Peking University Beijing China; ^4^ Department of Neurosurgery Shandong Provincial Hospital Affiliated to Shandong First Medical University Jinan China; ^5^ Key Laboratory of Traditional Chinese Medicine Classical Theory, Ministry of Education Shandong University of Traditional Chinese Medicine Jinan China; ^6^ Department of Neurology and Neurological Science Institute of Science Tokyo Tokyo China; ^7^ School of Traditional Chinese Medicine Jinan University Guangzhou China

**Keywords:** α‐Syn, antisense oligonucleotide, BDNF–TrkB, neuroprotective, Parkinson's disease

## Abstract

Parkinson's disease (PD) is characterized by the accumulation of Lewy bodies (LBs) in the brain, predominantly consisting of aggregated and phosphorylated α‐synuclein (α‐Syn). Consequently, strategies to suppress α‐Syn expression offer substantial therapeutic potential for PD. This study explores heteroduplex oligonucleotides (HDOs), an innovative gene‐silencing technology utilizing DNA/RNA or DNA/DNA hybrids. We designed two HDOs targeting α‐Syn: α‐Syn‐HDO‐1 (DNA/RNA) and α‐Syn‐HDO‐2 (DNA/DNA). Both demonstrated robust silencing of α‐Syn expression in mice. In AAV‐hSyn‐human SNCA‐treated mice, these α‐Syn‐HDOs exerted neuroprotective effects on dopaminergic neurons. Furthermore, they reduced aberrant α‐Syn accumulation in α‐Syn‐HEK293 cells and in mice treated with α‐Syn preformed fibrils (PFFs). The neuroprotective effects of α‐Syn‐HDOs were linked to activation of the brain‐derived neurotrophic factor (BDNF)‐tyrosine kinase B (TrkB) signaling pathway. Moreover, BDNF overexpression diminished abnormal α‐Syn aggregation in both PFFs‐treated α‐Syn‐HEK293 cells and PFFs‐treated mice. These results highlight the capacity of α‐Syn‐HDOs to protect dopaminergic neurons by enhancing BDNF–TrkB signaling through the inhibition of abnormal elevated α‐Syn. Our findings position α‐Syn‐HDOs as promising therapeutic candidates for PD and suggest BDNF as a potential diagnostic biomarker or indicator of treatment efficacy.

## Graphical abstract

α‐Syn‐HDOs protect dopaminergic neurons by enhancing BDNF–TrkB signaling through the inhibition of abnormally elevated α‐Syn. These findings position α‐Syn‐HDOs as promising therapeutic candidates for PD and suggest that BDNF may serve as a potential diagnostic biomarker or indicator of treatment efficacy.

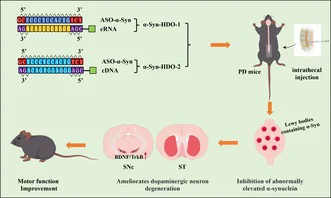

## 1 Introduction

Parkinson's disease (PD) is characterized by the progressive degeneration of dopaminergic neurons in the substantia nigra pars compacta (SNc), leading to the concurrent loss of nigrostriatal dopaminergic terminals and the onset of motor symptoms. PD is the second most common neurodegenerative disorder associated with aging (Kang et al. 2017b). Although the precise etiology of PD remains unclear, both its sporadic and familial forms share similar pathological features, notably the degeneration of dopaminergic neurons in the SNc and the presence of Lewy bodies (LBs), proteinaceous intraneuronal cytoplasmic inclusions in the surviving neurons (Dauer and Przedborski 2003; Pu et al. 2020). Alpha‐synuclein (α‐Syn) is the principal component of LBs, and its aggregation is central to PD pathogenesis (Volpicelli‐Daley et al. 2011). The phosphorylation of α‐Syn at the serine 129 site, a key post‐translational modification, promotes the formation of pathogenic α‐Syn aggregates (Zhang et al. 2019). Furthermore, both sporadic and familial forms of PD are linked to mutations or multiplications of the α‐Syn gene (Ellis et al. 2001; Jiang et al. 2013; Okochi et al. 2000). Targeting the inhibition of abnormal α‐Syn expression using antisense oligonucleotides has shown promise in improving PD pathology in mouse models (Cao et al. 2022; Yang et al. 2021). Therefore, the aberrant accumulation of α‐Syn contributes significantly to the vulnerability of dopaminergic neurons in both familial and sporadic PD, making it a potential therapeutic target for PD treatment.

DNA/RNA heteroduplex oligonucleotides (HDOs) represent a newly developed technology for gene silencing (Asada et al. 2021; Nishina et al. 2015; Yoshioka et al. 2019). Compared to the parent single‐stranded gapmer antisense oligonucleotides (ASOs), DNA/locked nucleic acid (LNA) gapmer duplexes with α‐tocopherol or cholesterol‐conjugated complementary RNA demonstrate significantly higher potency in reducing the expression of targeted mRNA while minimizing side effects (Nagata et al. 2021; Nishina et al. 2015; Yoshioka et al. 2019). Recent reports have shown that the complementary RNA strand in an HDO can be replaced with complementary DNA, and that DNA/DNA double‐stranded oligonucleotides are similarly effective in silencing targeted genes (Asami et al. 2021). In this study, we designed α‐Syn‐HDO‐1 (cholesterol‐conjugated complementary RNA) and α‐Syn‐HDO‐2 (cholesterol‐conjugated complementary DNA) specifically targeting α‐Syn. We evaluated the silencing effects of these α‐Syn‐HDOs on α‐Syn expression in mice and their neuroprotective effects on dopaminergic neurons in a PD mouse model.

This study reveals that α‐Syn‐HDO‐1 and α‐Syn‐HDO‐2 effectively suppressed α‐Syn expression in mice. In AAV‐hSyn‐human SNCA‐treated mice, both α‐Syn‐HDOs displayed neuroprotective effects on dopaminergic neurons. Additionally, these α‐Syn‐HDOs reduced aberrant α‐Syn accumulation in α‐Syn‐HEK293 cells and in mice treated with α‐Syn preformed fibrils (PFFs). The neuroprotective benefits of α‐Syn‐HDOs were linked to the activation of the brain‐derived neurotrophic factor (BDNF)‐tyrosine kinase B (TrkB) signaling pathway. Moreover, BDNF overexpression reduced abnormal α‐Syn accumulation in both PFFs‐treated α‐Syn‐HEK293 cells and PFFs‐treated mice.

## 2 Results

### 2.1 Both α‐Syn‐HDOs Silenced α‐Syn Expression in Mice

**FIGURE 1:**
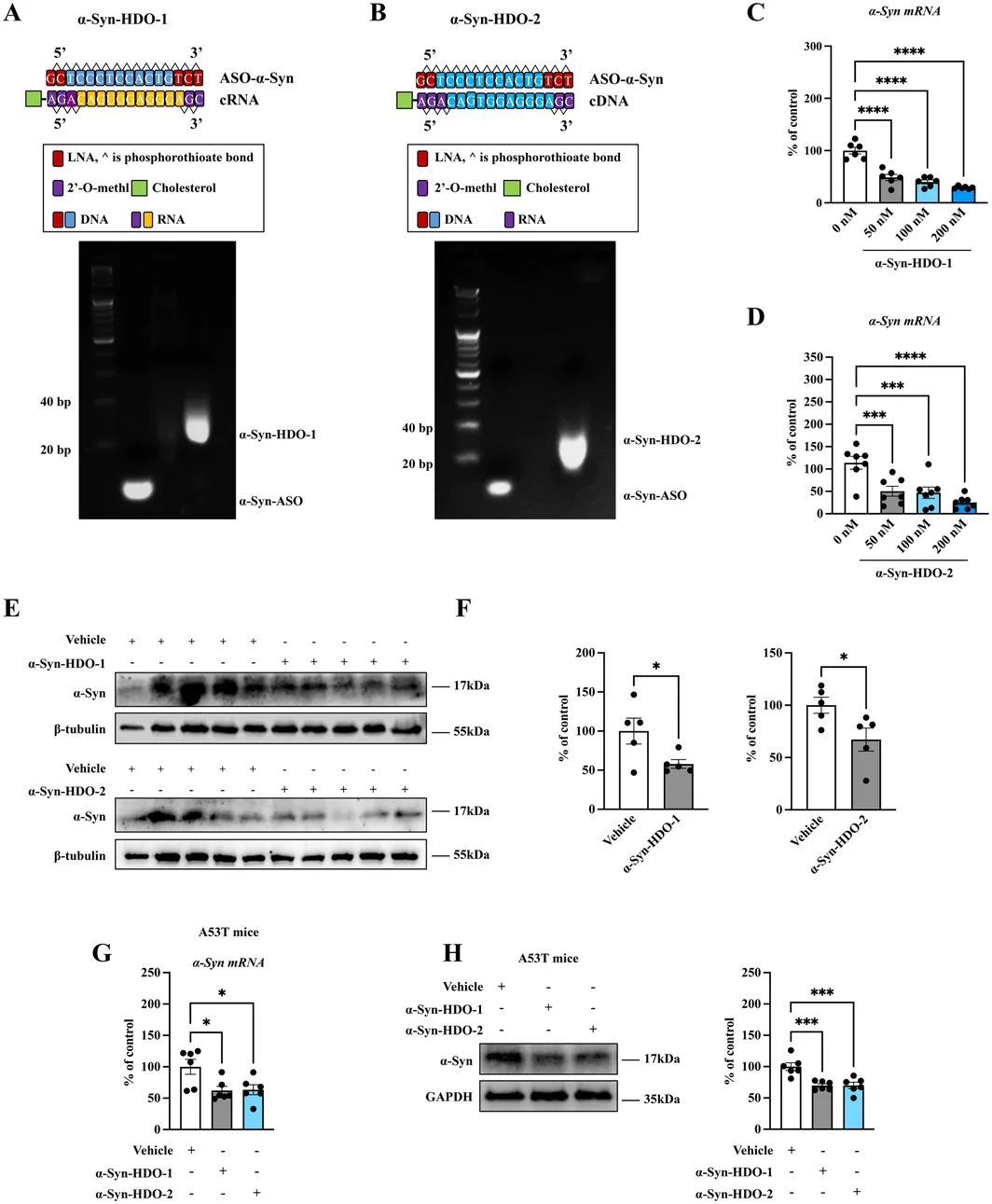
α‐Syn‐HDOs inhibit α‐Syn expression in mice. (A, B) Structure of α‐Syn‐HDO‐1 and α‐Syn‐HDO‐2, and confirm the successful synthesis of ASO and HDO. (C, D) The qPCR analysis of α‐Syn in the SNc of WT mice (mean ± SEM, *n* = 6 or 7 per group). (E, F) The representative image and quantification analysis of α‐Syn in the SNc of WT mice for western blot assay (mean ± SEM, *n* = 5 per group). (G, H) The qPCR and western blot analysis of α‐Syn in the SNc of A53T mice (mean ± SEM, *n* = 6 per group). *p < 0.05, ***p < 0.001, and ****p < 0.0001 compare with vehicle group, one‐way ANOVA.

We designed α‐Syn‐HDO‐1 and α‐Syn‐HDO‐2 to target α‐Syn and its complementary strand, each conjugated with a cholesterol ligand at the 5′ end. The formation of double‐stranded HDOs was confirmed by agarose gel electrophoresis (Figure 1A,B). Next, we assessed the silencing effects of these α‐Syn‐HDOs on α‐Syn expression. 72 h after intrathecal injection of α‐Syn‐HDOs, the SNc of WT mice was analyzed by qPCR and western blot. qPCR analysis revealed that both α‐Syn‐HDOs significantly reduced α‐Syn mRNA levels in the SNc of WT mice in a dose‐dependent manner (Figure 1C,D). Western blot analysis further demonstrated that both α‐Syn‐HDOs (200 nM/2 μL) significantly reduced α‐Syn protein levels in the SNc of WT mice (Figure 1E,F). The silencing effect of a single intrathecal injection persisted for at least 9 days (Figure S1A), and scrambled α‐Syn‐HDOs showed no silencing efficacy (Figure S1B). Furthermore, in A53T transgenic mice, α‐Syn‐HDOs significantly reduced human α‐Syn mRNA and protein levels in the SNc (Figure 1G,H). Collectively, these results demonstrate that both α‐Syn‐HDOs effectively silence mouse and human α‐Syn expression.

### 2.2 LRP1 and LDLR Facilitates the Uptake of α‐Syn‐HDOs in Dopaminergic Neurons

To evaluate the in vivo cellular uptake of α‐Syn‐HDO, we administered intrathecal injections of FAM‐labeled α‐Syn‐HDO‐1 (200 nM/2 μL) into WT mice for 12 and 24 h. Immunofluorescence staining revealed that TH and FAM signals colocalized in the SNc of mice (Figure S2A), indicating that α‐Syn‐HDO‐1 efficiently distributed within dopaminergic neurons. Next, to investigate the potential cell‐surface receptors facilitating α‐Syn‐HDO‐1 uptake, we noted that HDOs mediate effective gene suppression in both cardiac and skeletal muscle via a low‐density lipoprotein receptor (LDLR)‐dependent mechanism (Matsuda‐Kawabata et al. 2026). Given that α‐Syn‐HDO is conjugated to cholesterol, we focused on the LDLR and LDLR‐related protein 1 (LRP1) as potential candidates. 72 h after intrathecal injection of α‐Syn‐HDO‐1 (200 nM/2 μL), the SNc of WT mice was analyzed by qPCR and western blot. Both LRP1 antibody treatment and LRP1 inhibitor RAP significantly reduced the silencing effects of α‐Syn‐HDO‐1 on α‐Syn expression in the SNc (Figure S2B,C). Additionally, immunofluorescence staining with an anti‐LRP1 antibody and streptavidin–fluorescein isothiocyanate (FITC) demonstrated that biotinylated α‐Syn‐HDO‐1 (200 nM) colocalized with LRP1 in the cytoplasm of Neuro‐2a cells (Figure S2D). These results suggest that LRP1 may play a crucial role in facilitating the uptake of α‐Syn‐HDO‐1 into dopaminergic neurons.

For α‐Syn‐HDO‐2, WT mice were intrathecally injected with CY5‐labeled α‐Syn‐HDO‐2 (200 nM/2 μL) for 12 and 24 h. Immunofluorescence staining revealed that TH and CY5 signals colocalized in the SNc of mice (Figure S3A), indicating that α‐Syn‐HDO‐2 also efficiently distributed within dopaminergic neurons. Similar to the α‐Syn‐HDO‐1 experiments, SNc brain regions were collected 72 h after intrathecal injection of α‐Syn‐HDO‐2 (200 nM/2 μL) for further analysis. qPCR and western blot analyses demonstrated that treatment with LRP1 antibody, LDLR antibody, or RAP significantly inhibited the silencing effects of α‐Syn‐HDO‐2 on α‐Syn mRNA and protein levels in the SNc of WT mice (Figure S3B,C). Immunofluorescence staining with anti‐LDLR antibody, anti‐LRP1 antibody, and streptavidin–FITC revealed that biotinylated α‐Syn‐HDO‐2 (200 nM) colocalized with LDLR and LRP1 in the cytoplasm of Neuro‐2a cells (Figure S3D,E). These findings indicate that both LRP1 and LDLR mediate the cellular uptake of α‐Syn‐HDO‐2 in dopaminergic neurons.

### 2.3 α‐Syn‐HDOs Attenuate Dopaminergic Neuron Degeneration in AAV‐hSyn‐Human SNCA‐Treated Mice

**FIGURE 2:**
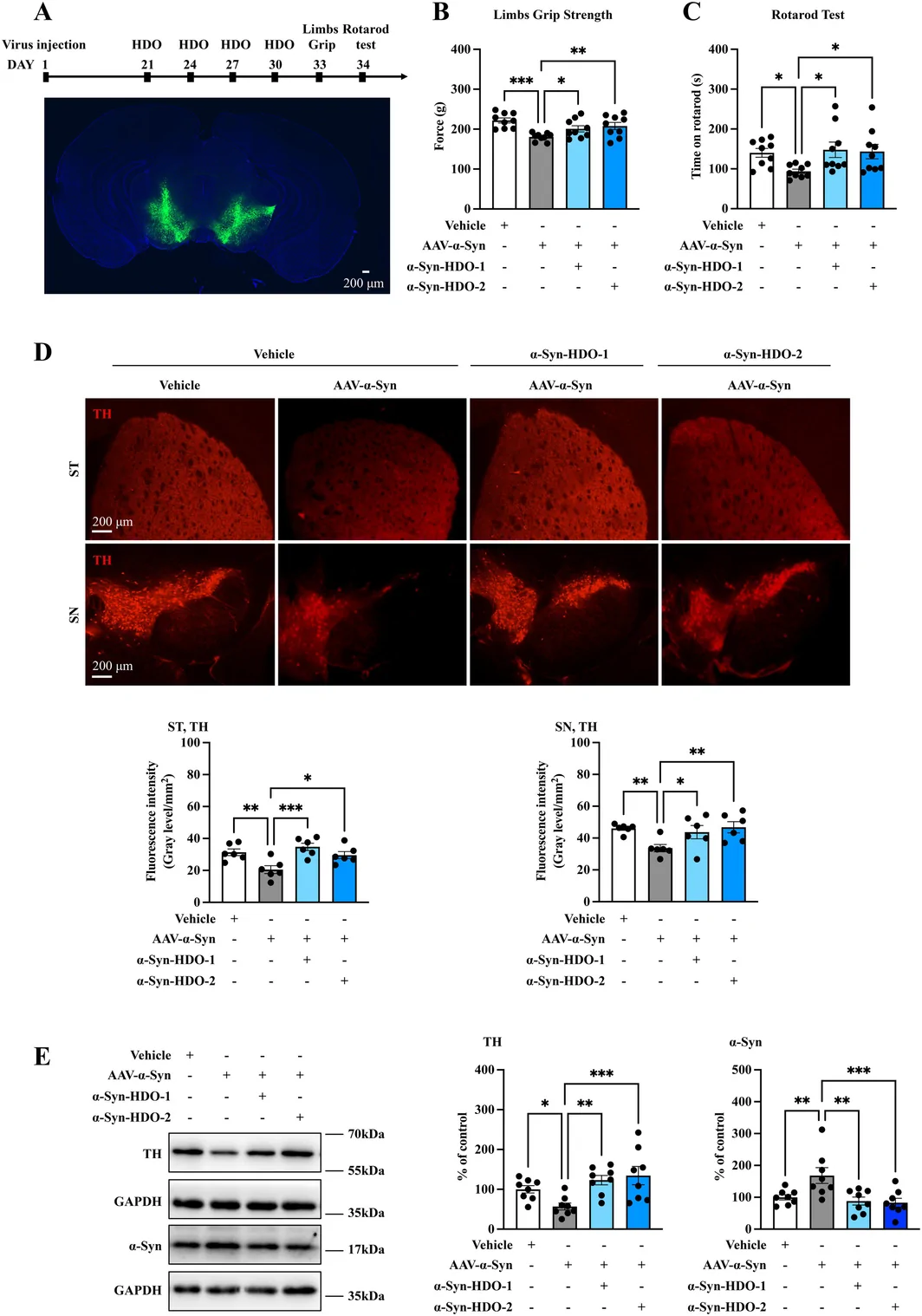
α‐Syn‐HDOs prevent AAV9‐hSyn‐human SNCA‐induced dopaminergic neurons degeneration in mice. (A) The schedule of treatments of AAV9‐hSyn‐human SNCA and the HDO and schematic diagram of the workflow for limbs grip strength test and rotarod test. Scale bar = 200 μm. (B, C) The limbs grip strength test and rotarod test (mean ± SEM, *n* = 9 per group). (D) The representative image and quantification analysis of TH in the striatum and SNc for immunofluorescence staining. Scale bar = 200 μm (mean ± SEM, *n* = 6 per group). (E) The quantification analysis of the TH and α‐Syn in the SNc for western blot assay (mean ± SEM, *n* = 8 per group). *p < 0.05, **p < 0.01, and ***p < 0.001 compare with AAV9‐hSyn‐human SNCA group, one‐way ANOVA.

To evaluate the therapeutic potential of α‐Syn‐HDOs in a PD mouse model, we injected AAV‐hSyn‐human SNCA into the SNc of mice to establish a PD mouse model (Figure 2A). Upon viral expression, the mice were intrathecally injected with α‐Syn‐HDOs (200 nM/2 μL) on Days 21, 24, 27, and 30, respectively (Figure 2A). Behavioral assessments were conducted on Days 33 and 34 (Figure 2A). In the grip strength test, both α‐Syn‐HDOs significantly improved grip strength in AAV‐hSyn‐human SNCA‐treated mice (Figure 2B). Similarly, in the rotarod test, α‐Syn‐HDOs significantly enhanced the duration of time spent on the rotating rod in these mice (Figure 2C). Immunofluorescence staining revealed that AAV‐hSyn‐human SNCA injection markedly reduced TH immunoreactivity in the striatum and SNc, but these effects were reversed by treatment with α‐Syn‐HDOs (Figure 2D). Western blot analysis showed that AAV‐hSyn‐human SNCA treatment significantly decreased TH expression while increasing α‐Syn levels in the SNc. These changes were reversed by both α‐Syn‐HDOs (Figure 2E). Collectively, these results suggest that these α‐Syn‐HDOs can attenuate dopaminergic neuron degeneration and alleviate PD‐like pathology in AAV‐hSyn‐human SNCA‐treated mice.

### 2.4 α‐Syn‐HDOs Inhibit Abnormal α‐Syn Aggregation In Vitro and In Vivo

Human α‐Syn PFFs induce the aggregation of endogenous α‐Syn in cultured cells, providing a valuable model for studying LBs pathology progression (Chmielarz and Domanskyi 2021). To evaluate the effects of α‐Syn‐HDOs on α‐Syn aggregation, we treated α‐Syn‐HEK293 cells with human PFFs. Following treatment, intracellular fluorescent puncta were observed, indicating α‐Syn aggregation (Figure S4A,B). Notably, both α‐Syn‐HDOs significantly attenuated this aggregation (Figure S4A,B). Western blot analysis confirmed that PFFs treatment increased p‐α‐Syn and α‐Syn levels in α‐Syn‐HEK293 cells, effects that were reversed by both α‐Syn‐HDOs (Figure S4C). To validate these findings in a more physiological context, we transduced primary neurons with human PFFs and observed that PFFs‐induced phosphorylation of endogenous α‐Syn was attenuated by α‐Syn‐HDOs (Figure S4D). Furthermore, in A53T mice, α‐Syn‐HDOs reduced the elevated p‐α‐Syn and α‐Syn levels in the SNc induced by human PFFs treatment (Figure S4E). Collectively, these results demonstrate that α‐Syn‐HDOs effectively counteract abnormal α‐Syn aggregation in PFFs‐treated α‐Syn‐HEK293 cells, primary neurons, and A53T mice.

### 2.5 α‐Syn‐HDOs Decrease Dopaminergic Neuronal Degeneration in PFFs‐Treated Mice

**FIGURE 3:**
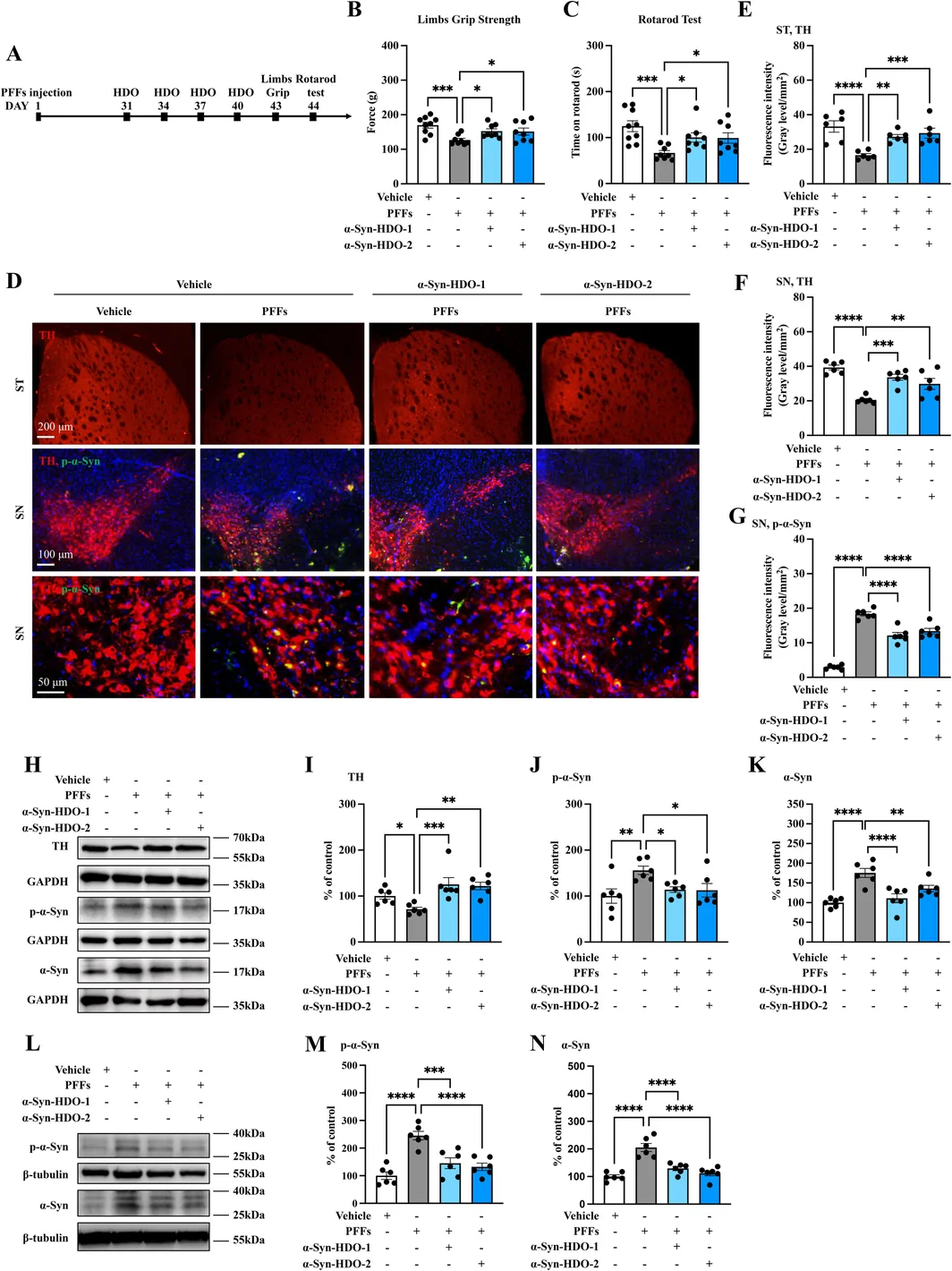
α‐Syn‐HDOs prevent PFFs‐induced dopaminergic neurons degeneration in mice. (A) The schedule of treatments of PFFs and the HDO and schematic diagram of the workflow for limbs grip strength test and rotarod test. (B, C) The limbs grip strength test and rotarod test (mean ± SEM, *n* = 8 or 9 per group). (D–G) The representative image and quantification analysis of TH and p‐α‐Syn in the striatum and SNc for immunofluorescence staining. Scale bar = 200 μm, 100 µm, and 50 µm (mean ± SEM, *n* = 6 per group). (H–N) The quantification analysis of the TH, p‐α‐Syn, and α‐Syn in the SNc for western blot assay (mean ± SEM, *n* = 5 or 6 per group). *p < 0.05, **p < 0.01, ***p < 0.001, and ****p < 0.0001 compare with PFFs group, one‐way ANOVA.

To determine whether α‐Syn‐HDOs mitigate α‐Syn aggregation in vivo by reducing α‐Syn expression, we evaluated their effects in a mouse model of PD induced by mouse PFFs (Sun et al. 2022) (Figure 3A). In the grip strength test, both α‐Syn‐HDOs significantly enhanced grip strength in PFFs‐treated mice (Figure 3B). Similarly, in the rotarod test, the α‐Syn‐HDOs significantly improved the duration time in PFFs‐treated mice (Figure 3C). Immunofluorescence staining showed that PFFs administration significantly decreased TH immunoreactivity in the striatum and SNc, while increasing p‐α‐Syn immunoreactivity in the SNc. These effects were attenuated by treatment with α‐Syn‐HDOs (Figure 3D–G). Western blot analysis demonstrated that PFFs treatment downregulated TH expression and increased levels of monomeric and high‐molecular‐weight oligomers of both p‐α‐Syn and α‐Syn in the SNc, effects that were reversed by both α‐Syn‐HDOs (Figure 3H–N). Collectively, these data demonstrate that α‐Syn‐HDOs inhibit the abnormal aggregation of α‐Syn in the SNc of PFFs‐treated mice, thereby mitigating dopaminergic neurodegeneration.

### 2.6 BDNF–TrkB Signaling Pathway Plays the Beneficial Role for the Neuroprotective Effects of α‐Syn‐HDOs

**FIGURE 4:**
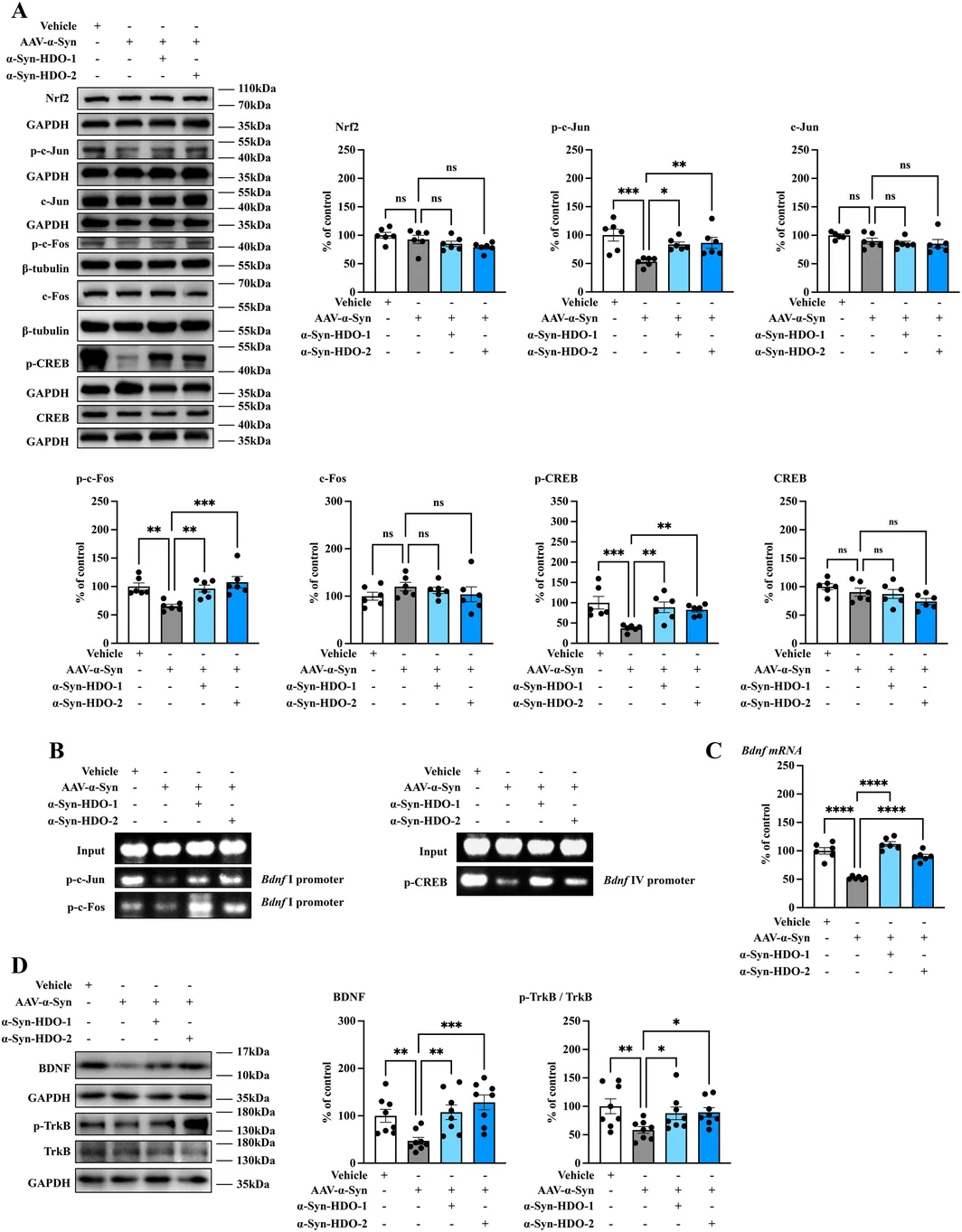
α‐Syn‐HDOs attenuated the inhibition of BDNF transcription in AAV9‐hSyn‐human SNCA‐treated mice. (A) The representative image and quantification analysis of Nrf2, p‐c‐Jun, c‐Jun, p‐c‐Fos, c‐Fos, p‐CREB, and CREB in the SNc for western blot assay (mean ± SEM, *n* = 6 per group). (B) The Chip‐PCR analysis of p‐c‐Jun, p‐c‐Fos, and p‐CREB for *Bdnf* exon promoter I and IV. (C) The qPCR analysis for BDNF in the SNc (mean ± SEM, *n* = 6 per group). (D) The representative image and quantification analysis of BDNF, p‐TrkB, and TrkB in the SNc for western blot assay (mean ± SEM, *n* = 8 or 9 per group). *p < 0.05, **p < 0.01, ***p < 0.001, and ****p < 0.0001 compared with AAV9‐hSyn‐human SNCA group, one‐way ANOVA.

Previous studies have established that suppressing aberrant α‐Syn expression via BDNF transcriptional activation mitigates dopaminergic neuron degeneration (Cao et al. 2022). Additionally, α‐Syn‐induced inhibition of TrkB neurotrophic signaling contributes to dopaminergic neuronal death in PD mouse models (Kang et al. 2017a). To elucidate the role of the BDNF–TrkB signaling pathway in the neuroprotective effects of α‐Syn‐HDOs, we evaluated BDNF transcription and TrkB phosphorylation status. Since the *Bdnf* exons I and IV promoters are primarily responsible for transcriptional control (Kidane et al. 2009; Rattiner et al. 2004; Tao et al. 2002), and given that Nrf2, c‐Jun, c‐Fos, and CREB regulate BDNF by binding to these promoters (Martinowich et al. 2003; Tang et al. 2021; Tuvikene et al. 2016), we focused our analysis on these key regulators. In the AAV‐hSyn‐human SNCA model, western blot analysis showed that α‐Syn‐HDOs significantly ameliorated the reduction in p‐c‐Jun, p‐c‐Fos, and p‐CREB levels, without affecting total Nrf2, c‐Jun, c‐Fos, or CREB expression in the SNc (Figure 4A). Chromatin immunoprecipitation (ChIP) assays demonstrated that α‐Syn‐HDOs reversed the dissociation of p‐c‐Jun, p‐c‐Fos, and p‐CREB from the *Bdnf* exon I or IV promoters (Figure 4B). Consequently, qPCR and western blot analyses revealed that α‐Syn‐HDOs restored the reduced *Bdnf* mRNA levels and normalized BDNF protein levels and the p‐TrkB/TrkB ratio in the SNc (Figure 4C,D).

**FIGURE 5:**
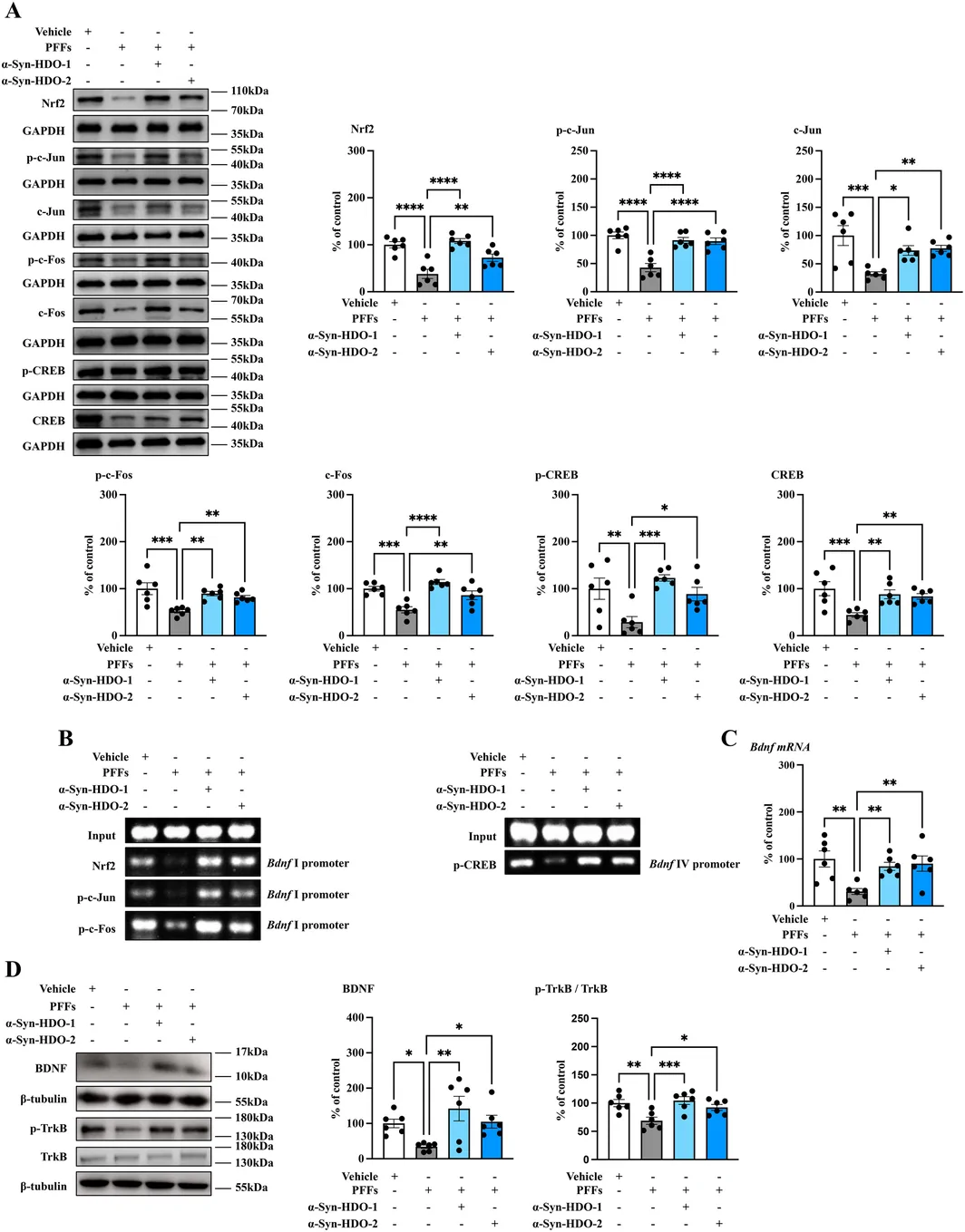
α‐Syn‐HDOs attenuated the inhibition of BDNF transcription in PFFs‐treated mice. (A) The representative image and quantification analysis of Nrf2, p‐c‐Jun, c‐Jun, p‐c‐Fos, c‐Fos, p‐CREB, and CREB in the SNc for western blot assay (mean ± SEM, *n* = 6 per group). (B) The Chip‐PCR analysis of Nrf2, p‐c‐Jun, p‐c‐Fos, and p‐CREB for *Bdnf* exon promoter I and IV. (C) The qPCR analysis for BDNF in the SNc (mean ± SEM, *n* = 6 per group). (D) The representative image and quantification analysis of BDNF, p‐TrkB, and TrkB in the SNc for western blot assay (mean ± SEM, *n* = 5 or 6 per group). *p < 0.05, **p < 0.01, ***p < 0.001, and ****p < 0.0001 compared with PFFs group, one‐way ANOVA.

Similarly, in the PFFs‐treated model, western blotting indicated that α‐Syn‐HDOs significantly rescued the reduction in Nrf2, p‐c‐Jun, c‐Jun, p‐c‐Fos, c‐Fos, p‐CREB, and CREB expression in the SNc (Figure 5A). ChIP assays confirmed that α‐Syn‐HDOs reversed the dissociation of Nrf2, p‐c‐Jun, p‐c‐Fos, and p‐CREB from the *Bdnf* exon I or IV promoters (Figure 5B). Furthermore, α‐Syn‐HDOs restored *Bdnf* mRNA levels (Figure 5C) and reversed the reduction in BDNF protein levels and the p‐TrkB/TrkB ratio (Figure 5D). Collectively, these findings indicate that α‐Syn‐HDOs alleviate BDNF transcriptional inhibition and activate the TrkB signaling pathway, thereby exerting neuroprotective effects in PD mouse models.

### 2.7 BDNF IV‐HDO Attenuates the Neuroprotective Effects of α‐Syn‐HDOs in AAV‐hSyn‐Human SNCA‐Treated Mice

**FIGURE 6:**
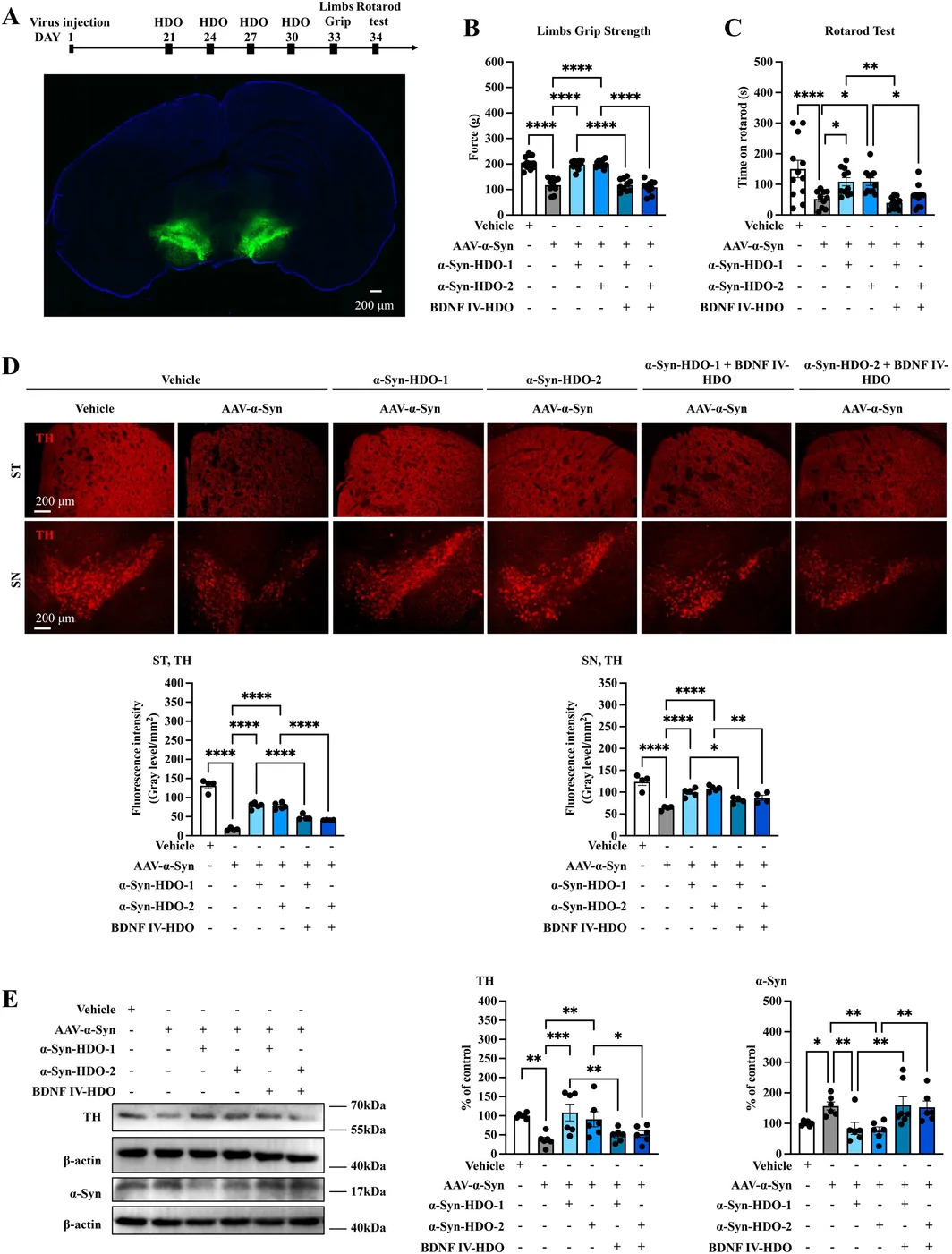
BDNF IV‐HDO attenuates the neuroprotective effects of α‐Syn‐HDOs in AAV9‐hSyn‐human SNCA‐treated mice. (A) The schedule of treatments of AAV9‐hSyn‐human SNCA and the HDO and schematic diagram of the workflow for limbs grip strength test and rotarod test. Scale bar = 200 μm. (B, C) The limbs grip strength test and rotarod test (mean ± SEM, *n* = 10–12 per group). (D) The representative image and quantification analysis of TH and α‐Syn in the striatum and SNc for immunofluorescence staining. Scale bar = 200 μm (mean ± SEM, *n* = 4 or 5 per group). (E) The quantification analysis of the TH and α‐Syn in the SNc for western blot assay (mean ± SEM, *n* = 6 per group). *p < 0.05, **p < 0.01, ***p < 0.001, and ****p < 0.0001 compared with AAV9‐hSyn‐human SNCA group or AAV9‐hSyn‐human SNCA + α‐Syn‐HDO‐1 group or AAV9‐hSyn‐human SNCA + α‐Syn‐HDO‐2 group, one‐way ANOVA.

To further validate the role of the BDNF–TrkB signaling pathway in the neuroprotective effects of these α‐Syn‐HDOs, we employed BDNF IV‐HDO (100 nM/2 μL) to inhibit BDNF expression (Yao et al. 2022) and evaluated the impact of this inhibition on the neuroprotective properties of α‐Syn‐HDOs. In the grip strength test, BDNF IV‐HDO significantly reduced the enhanced grip strength observed in α‐Syn‐HDOs‐treated mice that also received AAV‐hSyn‐human SNCA (Figure 6A,B). Similarly, in the rotarod test, BDNF IV‐HDO markedly reduced the improved duration time observed in these mice (Figure 6A,C). Immunofluorescence staining showed that BDNF IV‐HDO significantly diminished the increased TH immunoreactivity in the striatum and SNc of α‐Syn‐HDOs‐treated mice that received AAV‐hSyn‐human SNCA (Figure 6D). Additionally, Western blot analysis revealed that BDNF IV‐HDO significantly decreased TH expression and increased α‐Syn expression in the SNc of these mice (Figure 6E). These findings suggest that the neuroprotective effects of α‐Syn‐HDOs in AAV‐hSyn‐human SNCA‐treated mice are mediated, at least in part, through the BDNF–TrkB signaling pathway.

### 2.8 BDNF IV‐HDO Attenuates the Neuroprotective Effects of α‐Syn‐HDOs in PFFs‐Treated Mice

**FIGURE 7:**
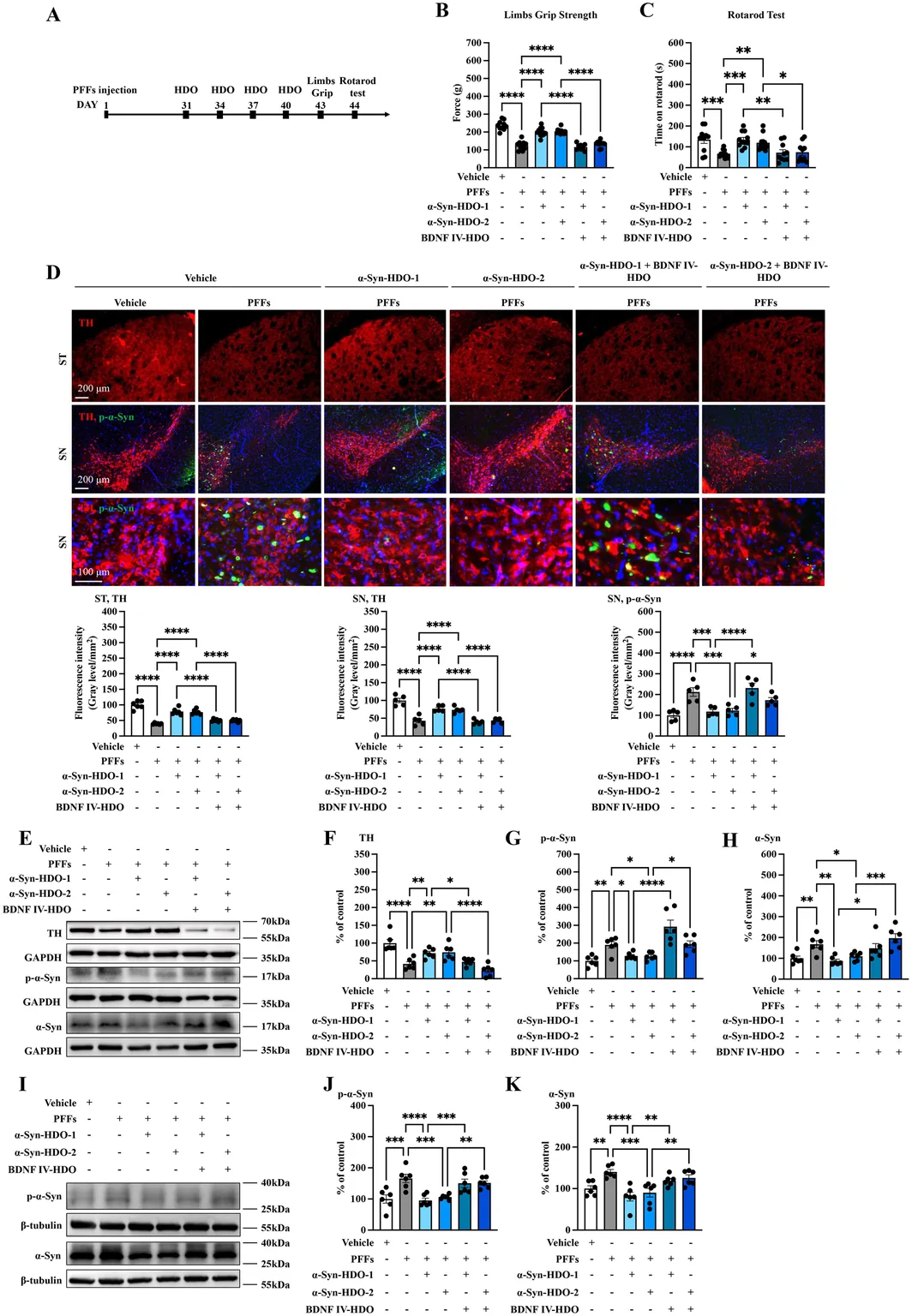
BDNF IV‐HDO attenuates the neuroprotective effects of α‐Syn‐HDOs in PFFs‐treated mice. (A) The schedule of treatments of PFFs and the HDO and schematic diagram of the workflow for limbs grip strength test and rotarod test. (B, C) The limbs grip strength test and rotarod test (mean ± SEM, *n* = 10–11 per group). (D) The representative image and quantification analysis of TH and α‐Syn in the striatum and SNc for immunofluorescence staining. Scale bar = 200 μm and 100 µm (mean ± SEM, *n* = 5 or 6 per group). (E–K) The quantification analysis of the TH, p‐α‐Syn, and α‐Syn in the SNc for western blot assay (mean ± SEM, *n* = 6 per group). *p < 0.05, **p < 0.01, ***p < 0.001, and ****p < 0.001 compared with PFFs group or PFFs + α‐Syn‐HDO‐1 group or PFFs + α‐Syn‐HDO‐2 group, one‐way ANOVA.

We further examined the impact of BDNF IV‐HDO on the neuroprotective effects of these α‐Syn‐HDOs in PFFs‐treated mice. In the grip strength test, BDNF IV‐HDO significantly diminished the improved grip strength observed in α‐Syn‐HDOs‐treated mice following PFFs administration (Figure 7A,B). Similarly, in the rotarod test, BDNF IV‐HDO markedly reduced the extended duration time in α‐Syn‐HDOs‐treated mice following PFFs administration (Figure 7A,C). Immunofluorescence staining revealed that BDNF IV‐HDO significantly attenuated the increased TH immunoreactivity in both the striatum and SNc, as well as the reduced p‐α‐Syn immunoreactivity in the SNc of α‐Syn‐HDOs‐treated mice following PFFs administration (Figure 7D). Likewise, western blot analysis demonstrated that BDNF IV‐HDO significantly downregulated TH expression and upregulated the levels of monomeric and high‐molecular‐weight oligomers of both p‐α‐Syn and α‐Syn in the SNc of these mice (Figure 7E–K). These findings indicate that the BDNF–TrkB signaling pathway mediates the neuroprotective effects of α‐Syn‐HDOs in PFFs‐treated mice.

### 2.9 BDNF IV‐HDO Attenuates the Neuroprotective Effects of α‐Syn‐HDOs by Inhibiting BDNF–TrkB Signaling Pathway

**FIGURE 8:**
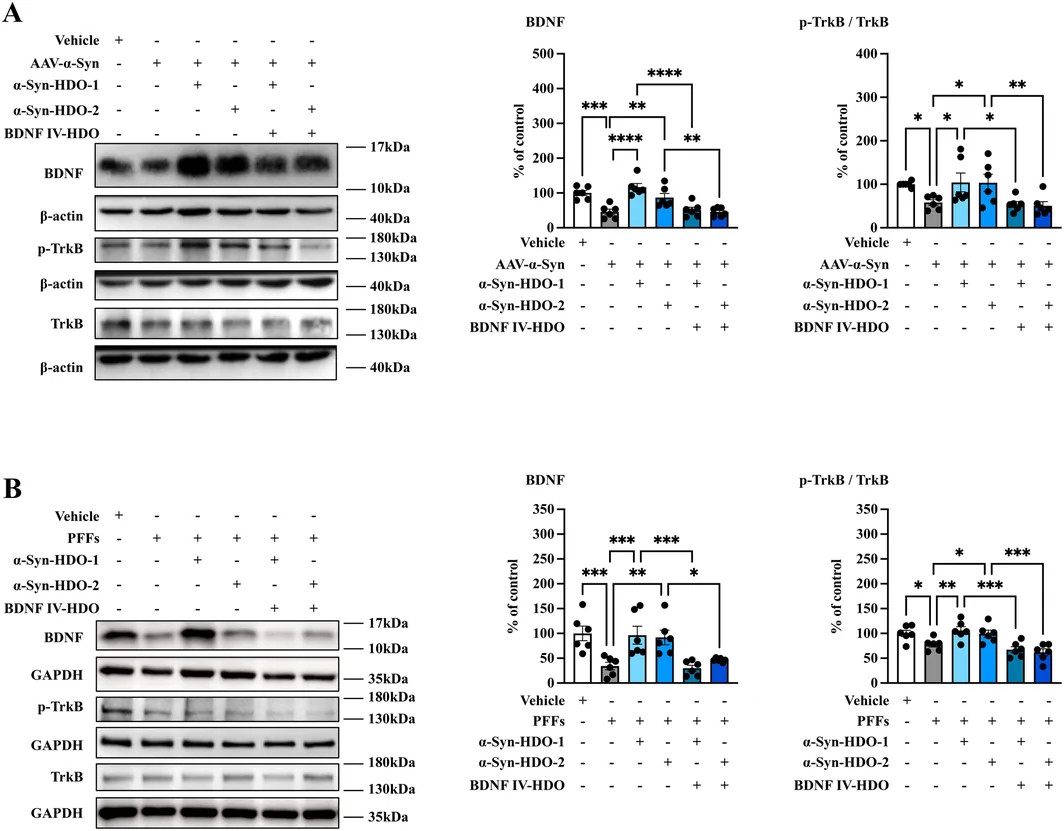
BDNF IV‐HDO attenuates the neuroprotective effects of α‐Syn‐HDOs by inhibiting the BDNF–TrkB signaling pathway. (A) The representative image and quantification analysis of BDNF, p‐TrkB, and TrkB in the SNc for western blot assay (mean ± SEM, *n* = 8 or 9 per group). (B) The representative image and quantification analysis of BDNF, p‐TrkB, and TrkB in the SNc for western blot assay (mean ± SEM, *n* = 5 or 6 per group). *p < 0.05, **p < 0.01, ***p < 0.001, and ****p < 0.0001 compared with PFFs group or α‐Syn‐HDO‐1‐ and α‐Syn‐HDO‐2‐treated mice following PFFs administration injection, one‐way ANOVA.

To further confirm the role of the BDNF–TrkB signaling pathway in the neuroprotective effects of α‐Syn‐HDOs, we analyzed BDNF protein levels and the ratio of p‐TrkB/TrkB in these mice. The findings revealed that BDNF IV‐HDO significantly decreased BDNF levels and the p‐TrkB/TrkB ratio in the SNc of α‐Syn‐HDOs‐treated mice that had also received AAV‐hSyn‐human SNCA (Figure 8A). Similarly, BDNF IV‐HDO notably reduced both BDNF levels and the p‐TrkB/TrkB ratio in the SNc of α‐Syn‐HDOs‐treated mice following treatment with PFFs (Figure 8B). These findings indicate that the neuroprotective effects of α‐Syn‐HDOs are dependent on the BDNF–TrkB signaling pathway.

### 2.10 BDNF Inhibits Aberrant α‐Syn Aggregation In Vitro

Our study demonstrated that reducing abnormally elevated α‐Syn expression decreases dopaminergic neuronal degeneration by increasing BDNF expression. To investigate BDNF's effect on α‐Syn aggregation, we treated α‐Syn‐HEK293 cells, transfected with TrkB and exposed to human PFFs, with BDNF protein (Figure S5A). Results showed that BDNF significantly reduced intracellular fluorescence spots, indicating inhibition of aberrant α‐Syn aggregation (Figure S5A). Western blot analysis confirmed that BDNF treatment markedly decreased levels of p‐α‐Syn and α‐Syn in these cells (Figure S5B).

Furthermore, the TrkB antagonist ANA‐12 blocked BDNF's inhibitory effects on aberrant α‐Syn aggregation (Figure S6A), while the TrkB agonist 7,8‐dihydroxyflavone (7,8‐DHF) similarly inhibited aggregation (Figure S6A). Western blot analysis revealed that ANA‐12 reversed BDNF's effects, increasing p‐α‐Syn and α‐Syn levels, and decreasing the p‐TrkB/TrkB ratio (Figure S6B). Conversely, 7,8‐DHF reduced p‐α‐Syn and α‐Syn levels, and elevated the p‐TrkB/TrkB ratio (Figure S6B). These findings suggest that BDNF effectively counteracts aberrant α‐Syn aggregation in PFFs‐treated α‐Syn‐HEK293 cells transfected with TrkB, likely through activation of the TrkB signaling pathway.

### 2.11 AAV‐hSyn‐Mouse BDNF Inhibits Dopaminergic Neuronal Degeneration in PFFs‐Treated Mice

Our in vitro studies demonstrated that BDNF reduces α‐Syn aggregation via activation of the TrkB signaling pathway. Here, we investigated whether BDNF exerts similar anti‐aggregation effects in PFFs‐treated mice and whether these effects are mediated by TrkB signaling. First, we examined the relationship between BDNF and p‐α‐Syn in SNc samples from PFFs‐treated mice. ELISA results showed elevated p‐α‐Syn levels and reduced BDNF levels in the SNc of PFFs‐treated mice, which were reversed by α‐Syn‐HDOs (Figure S7A,B). Furthermore, BDNF IV‐HDO attenuated the decreased p‐α‐Syn and the increased BDNF levels in the SNc of PFFs + α‐Syn‐HDOs‐treated mice (Figure S7C,D). These findings indicated a negative correlation between BDNF and p‐α‐Syn levels.

**FIGURE 9:**
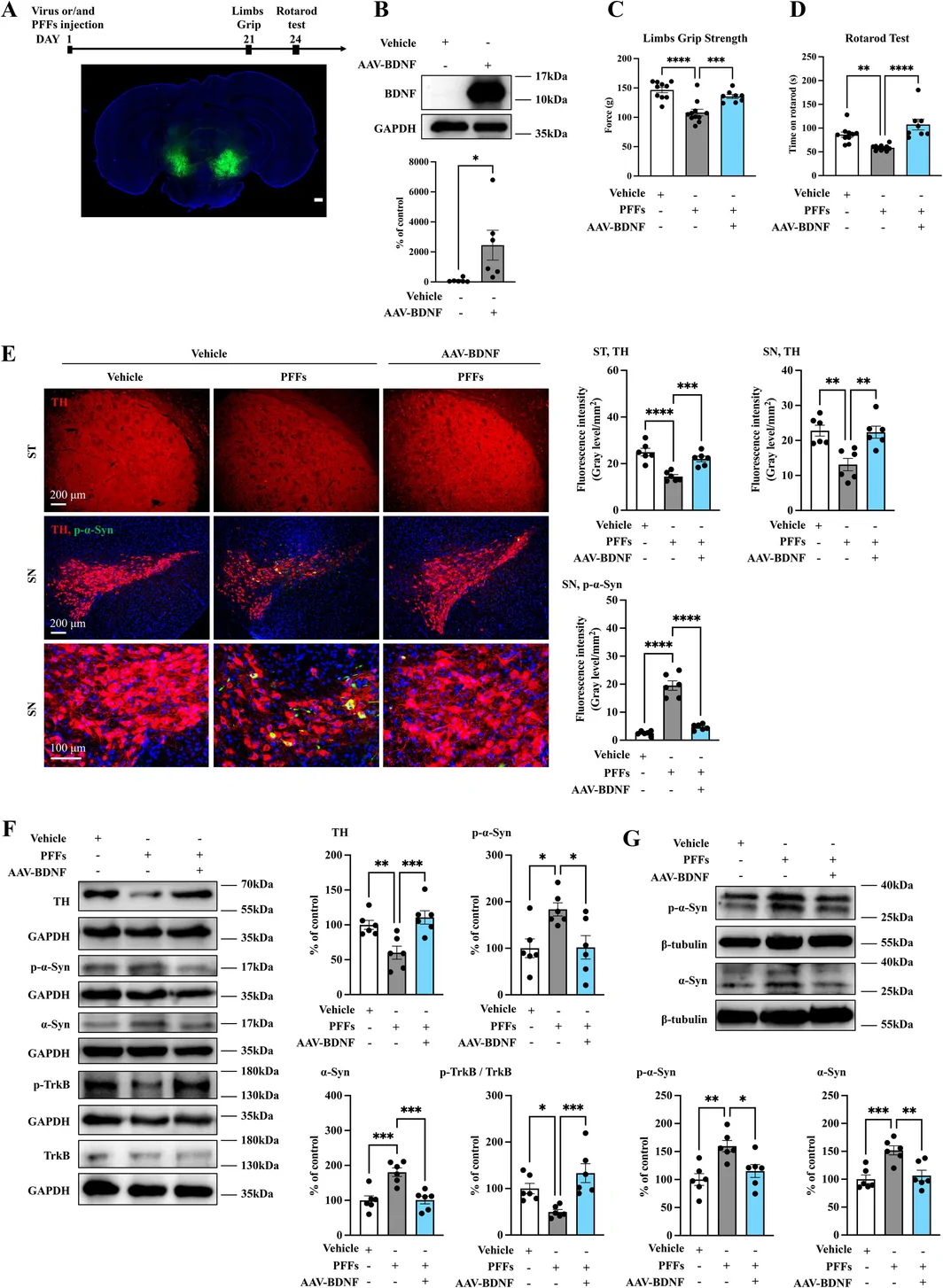
AAV‐hSyn‐mouse BDNF prevents PFFs‐induced dopaminergic neuron degeneration in mice. (A) Schedule of treatments of PFFs and the BDNF virus and schematic diagram of the workflow for limbs grip strength test and rotarod test. Scale bar = 200 μm. (B) The quantification analysis of BDNF in the SNc for western blot assay (mean ± SEM, *n* = 6 per group). *p < 0.05 compared with vehicle group, student *t* test. (C, D) The limbs grip strength test and rotarod test (mean ± SEM, *n* = 8–11 per group). (E) The representative image and quantification analysis of TH and p‐α‐Syn in the striatum and SNc for immunofluorescence staining. Scale bar = 200 μm and 100 µm (mean ± SEM, *n* = 6 per group). (F, G) The quantification analysis of the TH, p‐α‐Syn, α‐Syn, and the ratio of p‐TrkB/TrkB in the SNc for western blot assay (mean ± SEM, *n* = 6 per group). *p < 0.05, **p < 0.01, ***p < 0.001, and ****p < 0.0001 compared with PFFs group, one‐way ANOVA.

Next, we injected AAV‐hSyn‐mouse BDNF virus, with or without PFFs, into the SNc of mice and conducted behavioral tests 30 days post‐injection (Figure 9A,B). In the grip strength test, the BDNF virus significantly improved muscle strength in PFFs‐treated mice (Figure 9C). Similarly, in the rotarod test, the BDNF virus significantly prolonged latency‐to‐fall times in these mice (Figure 9D). Immunofluorescence staining revealed that the BDNF virus restored TH immunoreactivity in the striatum and SNc while reducing p‐α‐Syn immunoreactivity in the SNc of PFFs‐treated mice (Figure 9E). Western blot analysis confirmed that the BDNF virus increased TH levels and the p‐TrkB/TrkB ratio, while decreasing monomeric and high‐molecular‐weight oligomers of both p‐α‐Syn and α‐Syn in the SNc (Figure 9F,G). Finally, ELISA assays showed that the BDNF virus significantly decreased p‐α‐Syn and increased BDNF levels in the SNc of PFFs‐treated mice (Figure S7E,F). Collectively, these results demonstrate that BDNF overexpression attenuates dopaminergic neurodegeneration and alleviates PD‐like pathology in PFFs‐treated mice, likely through modulation of the TrkB signaling pathway.

## 3 Discussion

Our research demonstrates that both α‐Syn‐HDOs significantly reduce α‐Syn expression in mice. In the PD mouse model, both α‐Syn‐HDOs prevent dopaminergic neuron degeneration, an effect that appears to be mediated through activation of the BDNF–TrkB signaling pathway. Overexpression of BDNF diminished abnormal α‐Syn aggregation in both PFFs‐treated α‐Syn‐HEK293 cells and PFFs‐treated mice, which are associated with the TrkB signaling pathway.

LRP1 is an endocytic receptor that mediates the internalization of a diverse range of structurally and functionally distinct proteins (Gonias and Campana 2014). In certain cell types, LRP1‐mediated endocytosis is linked to activation of signaling pathways (Gonias and Campana 2014). In neurons, LRP1 facilitates the endocytosis of cholesterol‐containing lipoprotein particles, which are subsequently hydrolyzed in lysosomes (Liu et al. 2007). This process degrades apolipoprotein E (APOE) and releases free cholesterol for intracellular storage or lipoprotein assembly and membrane synthesis (Liu et al. 2007). Notably, deletion of LRP1 in forebrain neurons of adult mice significantly lowers brain cholesterol levels, highlighting its critical role in cerebral cholesterol metabolism (Liu et al. 2007). Since α‐Syn‐HDOs are linked to cholesterol, we suspected that LRP1 helps bring them into dopaminergic neurons. Supporting this, blocking LRP1 with antibodies or the inhibitor RAP reduced the ability of α‐Syn‐HDO‐1 to silence α‐Syn in the SNc of wild‐type mice. Also, staining showed that biotin‐labeled α‐Syn‐HDO‐1 and LRP1 are found together inside Neuro‐2a cells, suggesting LRP1 is important for α‐Syn‐HDO‐1 uptake. For α‐Syn‐HDO‐2, inhibiting LRP1 and LDLR with specific antibodies or the antagonist RAP reduced its silencing effect on α‐Syn mRNA and protein levels in the SNc. Immunofluorescence staining revealed that biotin‐labeled α‐Syn‐HDO‐2 co‐localizes with both LDLR and LRP1 in Neuro‐2a cells, suggesting that these receptors mediate its cellular uptake. However, we cannot rule out potential indirect effects resulting from receptor perturbation. Furthermore, given the greater structural complexity of α‐Syn‐HDO‐2 relative to α‐Syn‐HDO‐1, further studies are required to fully elucidate the mechanistic basis for these differences.

Numerous genetic and biochemical studies have established α‐Syn as a key component of LBs and a central factor in the pathogenesis of PD and DLB (Cao et al. 2022; Luk et al. 2012; Shahmoradian et al. 2019). Mutations or duplications of the α‐Syn gene are linked to both familial and sporadic forms of PD (Ellis et al. 2001; Jiang et al. 2013; Okochi et al. 2000). Soluble monomers, toxic oligomers, and insoluble fibrils of α‐Syn have been detected in PD patient brains (Lashuel et al. 2013). Several SNCA gene mutations—including A53T, A30P, E46K, H50Q, G51D, and A53E—cause autosomal dominant PD (Toffoli et al. 2020). One study showed that silencing abnormal α‐Syn expression with α‐Syn‐ASOs prevented α‐Syn synthesis and accumulation in connected brain regions, improving dopamine neurotransmission (Alarcon‐Aris et al. 2020). Another study demonstrated that α‐Syn‐ASOs attenuated α‐Syn aggregation induced by PFFs in vitro and in vivo, reducing dopaminergic neuron degeneration in A53T mutant mice (Yang et al. 2021). Furthermore, local α‐Syn‐ASO injection markedly decreased endogenous α‐Syn near the injection site. In a pathological propagation model, α‐Syn‐ASO injection at the seeding site prevented or ameliorated the initiation and spread of p‐α‐Syn pathology to both the injection site and connected brain regions over time, reducing associated pathologies (Sano et al. 2024). Although nucleic acid drugs targeting α‐Syn have been reported, safer and more effective therapies are still needed. HDOs represent a novel gene‐silencing technology (Asada et al. 2021; Nishina et al. 2015; Yoshioka et al. 2019). Compared to parent single‐stranded gapmer ASOs, DNA/LNA gapmer duplexes with cholesterol‐conjugated complementary RNA exhibit significantly greater potency in reducing target mRNA expression while minimizing side effects (Nagata et al. 2021). In our study, both α‐Syn‐HDO‐1 and α‐Syn‐HDO‐2 potently silenced α‐Syn in mice. Using an AAV‐hSyn‐human SNCA mouse model, both HDOs significantly reduced dopaminergic neuron degeneration and alleviated PD‐like pathology. These findings suggest that downregulating abnormal α‐Syn is a promising therapeutic strategy to halt PD progression.

Phosphorylation of α‐Syn at serine 129 (S129) is a hallmark of LBs in PD (Anderson et al. 2006). It is widely hypothesized that phosphorylation at Ser129 promotes the formation of insoluble α‐Syn aggregates, a key event in PD pathogenesis (Arawaka et al. 2017). Studies have shown that exogenous PFFs can induce aggregation of endogenous α‐Syn (Karampetsou et al. 2017; Luk et al. 2012). Therefore, we investigated whether α‐Syn‐HDOs could inhibit abnormal α‐Syn aggregation. Our results demonstrate that both α‐Syn‐HDOs reduced α‐Syn expression, phosphorylation, and aggregation in PFFs‐treated α‐Syn‐HEK293 cells and PFFs‐treated mice. Furthermore, α‐Syn‐HDOs alleviated dopaminergic neuron degeneration in PFFs‐treated mice. Given their ability to suppress phosphorylated α‐Syn (S129), these data suggest that α‐Syn‐HDOs may mitigate pathological changes by reducing abnormal α‐Syn levels.

BDNF, a key member of the neurotrophin (NT) family (Huang and Reichardt 2003; Kang et al. 2017a; Mizui et al. 2016; Zhang et al. 2016), is co‐colocalized with and promotes the survival of dopaminergic neurons in the SNc (Baydyuk and Xu 2014; Seroogy et al. 1994). Consistent with this, clinical studies have identified reduced BDNF levels in patients with PD, implicating this deficit in the disease's pathogenesis (Howells et al. 2000; Mogi et al. 1999). In clinically and neuropathologically typical PD, SNpc BDNF mRNA expression is reduced (Howells et al. 2000). This loss of nigral BDNF mRNA directly correlates with decreased neuronal soma size and survival, indicating that neurons with low BDNF expression are particularly vulnerable to degeneration (Howells et al. 2000). In line with this, serum BDNF levels are lower in early‐stage PD patients compared to healthy controls (Scalzo et al. 2010). However, this relationship becomes complex in later stages, where BDNF levels correlate positively with disease duration and severity, potentially reflecting a compensatory neuroprotective response (Scalzo et al. 2010). Mechanistically, pathological α‐Syn disrupts BDNF homeostasis through multiple pathways: targeted α‐Syn overexpression suppresses BDNF gene and protein expression and impairs downstream signaling (Fang et al. 2017; Wang et al. 2016; Yuan et al. 2010). Conversely, silencing α‐Syn leads to a marked upregulation of BDNF mRNA (Wang et al. 2016). Critically, α‐Syn overexpression also disrupts the retrograde transport of BDNF itself and its receptor, TrkB (Fang et al. 2017). Neurons harboring PFFs‐induced α‐Syn inclusions exhibit reduced TrkB transport, and α‐Syn has been shown to directly inhibit BDNF–TrkB signaling in vitro (Kang et al. 2017a). Collectively, these findings delineate a pathogenic cascade whereby pathological α‐Syn decreases BDNF production, impairs its retrograde transport, and diminishes TrkB receptor availability and signaling. This multifaceted disruption strongly suggests that augmenting BDNF expression or signaling represents a plausible therapeutic strategy to counteract the neurodegenerative consequences of synucleinopathy.

In the current study, we demonstrate that the BDNF–TrkB signaling pathway is the primary mediator of the neuroprotective effects of α‐Syn‐HDOs. Downregulation of BDNF attenuated the neuroprotective effects of α‐Syn‐HDOs in mice treated with AAV‐hSyn‐human SNCA and PFFs. Conversely, BDNF overexpression effectively attenuated aberrant α‐Syn aggregation in both PFFs‐treated α‐Syn‐HEK293 cells and PFFs‐treated mice. Furthermore, BDNF overexpression protected against dopaminergic neurons' degeneration in the PFFs mouse model. Pharmacological inhibition using ANA‐12 (a TrkB antagonist) successfully blocked BDNF's inhibitory effects on aberrant α‐Syn aggregation. In contrast, 7,8‐DHF (a TrkB agonist) replicated BDNF's effects by suppressing α‐Syn aggregation. These converging lines of evidence suggest that the neuroprotective effects of α‐Syn‐HDOs in the PD mouse model are dependent on the activation of the BDNF–TrkB signaling pathway.

This study has several limitations. Regarding the silencing of LRP1, we utilized an LRP1 antibody as the silencing agent. Although effective for our experimental aims, this approach cannot achieve complete gene knockdown. Future studies employing complementary genetic approaches, such as siRNA or CRISPR‐Cas9, will be essential to definitively validate our findings and fully elucidate LRP1's role. In addition, this study utilized only male mice. While female mice could also be considered, we speculate that α‐Syn‐HDOs may similarly alleviate PD‐like symptoms in females. However, this requires confirmation through further detailed studies.

In conclusion, our findings demonstrate that both α‐Syn‐HDOs effectively prevent dopaminergic neuron degeneration in the PD mouse model, an effect mediated through the BDNF–TrkB signaling pathway. These results highlight the potential of α‐Syn‐HDOs as novel therapeutic agents for PD and suggest that BDNF could serve as a diagnostic biomarker or an indicator of therapeutic efficacy.

## 4 Methods

### 4.1 Mice

Male A53T human α‐Syn mice (line M83, stock number 004479, Jackson Laboratory) and adult C57BL/6 mice (8 weeks old, weighing 20–25 g, Charles River) were used in the experiments. The animals were housed under controlled temperature conditions and maintained on a 12‐h light/dark cycle with ad libitum access to food and water. All animal procedures were approved by the Shandong First Medical University Institutional Animal Care and Use Committee, and the experiments were conducted in accordance with the Guide for Animal Experimentation of Shandong First Medical University (W202406130638).

### 4.2 Cell Lines, and Primary Neurons

The Neuro‐2a cells and HEK‐293 cells expressing YFP‐labeled human α‐Syn (α‐Syn‐HEK293 cells) are cultured in DMEM with 10% fetal bovine serum (Excell Bio.) and 1% penicillin (100 units/mL)–streptomycin (100 μg/mL) at 37°C in a 5% CO_2_ atmosphere, ensuring appropriate growth and stability for the cells in a humidified incubator.

Primary neurons were cultured as described previously (Kim et al. 2017; Lin et al. 2023; Wu et al. 2021). Briefly, the brains of newborn mice were dissected and incubated in cold Hank's Balanced Salt Solution (HBSS), free of Ca^2+^ and Mg^2+^ (4 mL HBSS per brain). The tissue was dissociated with 0.05% trypsin in HBSS for 10 min at 37°C, followed by centrifugation at 300 × *g* for 2 min. The resulting pellet was resuspended in minimal essential medium (MEM) containing 10% FBS, 10% heat‐inactivated horse serum, 2 mM L‐glutamine, and 1% penicillin–streptomycin. The cell suspension was filtered twice through a 70 μm cell strainer. Approximately 5 × 10^7^ neurons were isolated from 20 to 30 brains. Neurons were then plated onto poly‐D‐lysine‐coated plates at a concentration of 0.2 mg/mL and cultured in serum‐free Neurobasal medium, supplemented with 2% B27, 1% L‐glutamine, and 1% penicillin–streptomycin, and incubated at 37°C.

### 4.3 Materials

ASOs, cRNA, and cDNA targeting α‐Syn were obtained from TsingKe Biological Technology (Wuhan, China) and solubilized in 0.9% sterile saline prior to use. For the formation of α‐Syn‐HDOs, equimolar amounts of DNA and cRNA or cDNA strands were heated in 0.9% sterile saline at 95°C for 5 min and then gradually cooled to room temperature. The HDOs contained locked nucleic acids (LNAs) at each end flanking the central base of DNA, with or without a fluorescent label (FAM [6‐carboxy‐fluorescein], CY5 [Sulfo‐Cyanine5], or Biotin), or 2′‐O‐methyl modifications at each end flanking the central base of cRNA or cRNA‐cDNA hybrid strands, conjugated with cholesterol (Figure 1A,B). The sequences of the ASOs, cRNA, and cDNA used in these experiments are as follows: ASO‐α‐Syn: G(L)^C(L)^t^c^c^c^t^c^c^a^c^t^g^T(L)^C(L)^T(L) (Yang et al. 2021); cRNA, a(M)^g(M)^a(M)^caguggaggga^g(M)^c(M); cDNA, a(M)^g(M)^a(M)^cagtggaggga^g(M)^c(M). Scrambled ASO‐α‐Syn: A(L)^C(L) ^t^c^c^c^g^a^a^c^c^t^g^T(L)^C(L)^T(L) (Yang et al. 2021). cRNA: a(M)^g(M)^a(M)^cagguucgggag^(M)^t(M); BDNF exon IV‐ASO: C(L)^A(L)^G^T^C^A^C^T^A^C^T^T^G^T^C^A^A^A^G(L)^T(L)^A(L); BDNF exon IV‐cRNA: u(M)^a(M)^c(M)^uuugacaaguagugacu(M)^g(M) (Bambah‐Mukku et al. 2014). Where L indicates locked nucleic acids, M represents 2′‐O‐methyl modifications, and ^ denotes phosphorothioate bonds. α‐Syn‐HEK293 cells and primary neurons were transfected with α‐Syn‐HDO‐1 and α‐Syn‐HDO‐2 for 24 h using Lipofectamine 3000 (Invitrogen) according to the manufacturer's instructions. Following 24 h of transfection, cells were collected for immunofluorescence staining. Recombinant human receptor‐associated protein (RAP, 10 μM Molecular Innovations), N2‐(2‐ {[(2‐oxoazepan‐3‐yl) amino]carbonyl}phenyl)benzo[b]thiophene‐ 2‐carboxamide (ANA‐12, 10 nM, HY‐12497) (Cazorla et al. 2011), 7,8‐Dihydroxyflavone (7,8‐DHF, 0.5 μM, HY‐W013372) (Jang et al. 2010), and human BDNF protein (HY‐P7116A) were purchased from MedChemExpress.

### 4.4 Preparation of Preformed Fibrils (PFFs)

Full‐length human and mouse α‐Syn were expressed in BL21 (DE3) competent *E. coli* (Life Technologies) and purified as previously described (Volpicelli‐Daley et al. 2014). The purified recombinant α‐Syn was stored at −80°C until use. PFFs were prepared by diluting recombinant α‐Syn to 5 mg/mL in sterile Dulbecco's PBS (Cellgro, Mediatech; pH adjusted to 7.0, without Ca^2+^ or Mg^2+^), followed by incubation at 37°C with constant agitation at 1000 rpm for 7 days. The PFFs were then sonicated using a water‐bath cup‐horn sonicator (Fisher Scientific, USA) at 50% power for 5 min before use.

### 4.5 Treatment With AAV‐hSyn‐Human SNCA, AAV‐hSyn‐Mouse *Bdnf*, PFFs, α‐Syn‐HDOs, Low‐Density Lipoprotein Receptor–Related Protein‐1 (LRP1) Antibody, and Low‐Density Lipoprotein Receptor (LDLR) Antibody

Mice were anesthetized with isoflurane and secured in a stereotaxic apparatus. AAV‐hSyn‐human SNCA (6.58 × 10^13^ vg/mL, Sunbio Medical Biotechnology), or AAV‐hSyn‐mouse *Bdnf* (9.19 × 10^12^ vg/mL, Sunbio Medical Biotechnology), or PFFs (human or mouse) were injected into the substantia nigra (±1.2 mm lateral, −4.3 mm ventral, and −3.1 mm from Bregma) (Kang et al. 2017b). Virus (0.5 μL) or PFFs (5 μg) were injected into each site using a 10 μL Hamilton syringe with a fixed needle at a rate of 0.25 μL/min using a microinjector pump (KDS, Stoelting).

The intrathecal administration of α‐Syn‐HDOs was performed as previously described (Ferreira et al. 1999). Mice were anesthetized with isoflurane and received an injection of α‐Syn‐HDOs (50 nM, 100 nM, and 200 nM/2 μL), BDNF IV‐HDO (100 nM/2 μL) (Yao et al. 2022), or 0.9% sterile saline solution between the L5 and L6 vertebrae using a 10 μL Hamilton syringe. Following the injection, the animals were placed on a heating pad to provide thermal support until full recovery from anesthesia.

LRP1 and LDLR antibodies were administered via intracerebroventricular (ICV) injection. For α‐Syn‐HDO‐1‐treated mice, 0.066 μg of LRP1 and 2 μg of LDLR antibody were used for both quantitative real‐time PCR (qPCR) and western blot analyses. In α‐Syn‐HDO‐2‐treated mice, 0.066 μg of LRP1 and 2 μg of LDLR antibody were used for qPCR analysis, while 0.099 μg of LRP1 and 3 μg of LDLR antibody were used for western blot analysis.

### 4.6 Quantitative Real‐Time PCR (qPCR) Assay

Levels of α‐Syn mRNA were measured by qPCR. RNA was extracted using the Eastep Super Kit (Promega) and reverse transcribed into cDNA using the GoScript Reverse Transcriptase Mix with Oligo(dT) primers (Promega). Real‐time PCR was performed using the ChamQ SYBR qPCR Master Mix Kit (Vazyme) on a 788BR05175 Real‐Time PCR System. The PCR amplification conditions were as follows: 40 cycles of denaturation at 95°C for 30 s, annealing at 55°C for 30 s, and extension at 72°C for 30 s. The primer sequences were: human *α‐Syn* forward, 5′ TGACGGGTGTGACAGCAGTAG 3′; human *α‐Syn* reverse, 5′ CAGTGGCTGCTGCAATG 3′; mouse *α‐Syn* forward, 5′ CACTGGCTTTGTCAAGAAGGACC 3′; mouse *α‐Syn* reverse, 5′ CATAAGCCTCACTGCCAGGATC 3′.

### 4.7 Western Blot Analysis

Cell or brain homogenates were lysed in RIPA buffer, and protein concentrations were determined using a Coomassie Brilliant Blue protein assay kit (Bio‐Rad). Total protein (30 μg) was separated by SDS‐PAGE or Native‐PAGE (for high‐molecular‐weight of α‐Syn species) on 10%–12% gels and transferred to polyvinylidene difluoride (PVDF) membranes. The membranes were blocked with 5% milk at room temperature for 1 h, followed by incubation with primary antibodies at 4°C for 12 h. Membranes were then washed three times with TBST and incubated with the corresponding secondary antibody for 1 h at room temperature. After three additional washes, the targeted proteins were detected using an enhanced chemiluminescence method and scanned with a Tanon‐5200CE imaging system (Tanon, Shanghai, China). A portion of the target protein was washed by antibody clearing solution and subsequently incubated with antibodies to the target protein. The following primary antibodies were used: BDNF antibody (1:1000, ab108319), p‐α‐Syn antibody (1:1000, ab51253), α‐Syn antibody (1:1000, ab1903), and Nrf2 (1:1000, ab31163) from Abcam; p‐TrkB antibody (1:1000, AF3461), TrkB antibody (1:1000, 4603) from Cell Signaling Technology; p‐CREB antibody (1:1000, 9198S), CREB antibody (1:1000, 9197S), p‐c‐Jun antibody (1:1000, 9261S), and c‐Jun antibody (1:1000, 9165S) from Cell Signaling Technology; p‐c‐Fos antibody (1:1000, bs‐3154R) from Bioss, c‐Fos antibody (1:1000, 21667) from SAB; tyrosine‐hydroxylase (TH) antibody (1:1000, GTX634481) from GeneTex; β‐tubulin (1:1000, AF7011) and GAPDH (1:1000, AF7021) antibodies from Affinity; and HRP‐conjugated anti‐rabbit/mouse IgG antibody from Bio‐Rad.

### 4.8 Immunofluorescence Staining

The cells or brain sections from mice were placed on glass slides and fixed with 4% paraformaldehyde (PFA) at room temperature for 10 min. The slides were washed three times with PBS, followed by blocking with 3% BSA and 0.3% Triton X‐100 for 30 min. The samples were then incubated with primary antibodies against TH (GTX634481 and AF6113, 1:500), p‐α‐Syn (ab51253, 1:500), α‐Syn (ab1903, 1:500), low‐density lipoprotein receptor (LDLR) (Affinity #DF7696, 1:500), LDLR‐related protein 1 (LRP1) (CST, 64099, 1:500), and MAP2 (Proteintech 67015‐1‐Ig, 1:500) for 48 h at 4°C. The cells and brain sections were then washed with PBS and incubated with Alexa Fluor 488/594 anti‐mouse/rabbit secondary antibody (1:500) for 2 h at room temperature in the dark, followed by DAPI staining to visualize the nuclei. Visualization of cells and brain sections was performed using a fluorescence microscope (Olympus BX53, Japan). The striatum and substantia nigra from three brain sections per mouse were analyzed and averaged. Fluorescence intensity was quantified by first binarizing the images using a fixed brightness threshold (values 48–255) in ImageJ (version 2.1.0/1.53c), which was then used for baseline correction. All analyses were performed by investigators blinded to the treatment groups.

### 4.9 Enzyme‐Linked Immunosorbent Assay (ELISA)

Brain tissues were homogenized in PBS. Following homogenization, samples were centrifuged at 15,000 × *g* for 4 min at 4°C, and the resulting supernatant was collected. Supernatants were subsequently diluted 1:10 in ELISA diluent and analyzed using specific ELISA kits (Yuanju Bio, YJ338974). Supernatants levels of p‐α‐Syn and BDNF were also quantified by ELISA according to the manufacturer's instructions.

### 4.10 Behavioral Tests

For the grip strength test, each mouse's limbs were gently placed in the center of the grip plate to encourage grasping. The animal was then gently pulled backward by the tail. Each mouse was tested three times, and the average of the three measurements was used for analysis.

For the rotarod test, mice were trained for 3 consecutive days. Each training session involved placing the mouse on the rotarod at a slow rotational speed (5 rpm) for a maximum of 5 min. On the test day, mice underwent three trials, with the rotational speed increasing from 0 to 40 rpm at a rate of 0.1 rpm/s. Each trial lasted for 5 min or until the mouse fell, which activated a switch to automatically stop the timer. The residence time on the rotarod was measured with a stopwatch. Results were presented as the average of the three trials.

### 4.11 Statistical Analysis

All data are expressed as the mean ± standard error of the mean (SEM), calculated using PASW Statistics 26 software (R26.0.0.0, formerly SPSS Statistics, SPSS). Potential differences between means were assessed using one‐way analysis of variance (ANOVA), followed by post hoc Fisher's least significant difference (LSD) test. Student's *t*‐test was used to compare differences between two groups, unless otherwise specified. Statistical significance was indicated by asterisks: *p < 0.05, **p < 0.01, and ***p < 0.001. *p* values greater than 0.05 were considered non‐significant (ns).

## Author Contributions

X.W., Y.D., and L.H. performed the microinjection, behavior test, immunofluorescence staining assay, qPCR assay, and western blot assay; X.K. performed the cell culture. F.S., T.Y., and X.G. assisted in editing the manuscript. L.‐w.Z., W.Y., and J.‐c.Z. conceived of the project, designed the experiments, and wrote the manuscript. All authors approved the manuscript.

## Funding

This work was supported by National Natural Science Foundation of China (82271563).

## Conflicts of Interest

T.Y. has ongoing collaborations with Takeda Pharmaceutical Co. Ltd. and serves as an academic advisor for Rena Therapeutics Inc. The other authors declare no competing interests.

## Supporting information


**Figure S1:** α‐Syn‐HDOs exhibit a long‐term silencing effect on α‐Syn. (A) Representative western blot images and quantification of α‐Syn levels in the SNc following vehicle or α‐Syn‐HDOs injection (mean ± SEM, *n* = 6 per group). (B) Representative western blot images and quantification of α‐Syn levels in the SNc following vehicle or scrambled α‐Syn‐HDOs injection (mean ± SEM, *n* = 6 per group). *p < 0.05 and **p < 0.01 compared with vehicle group, one‐way ANOVA. ns, not significant.
**Figure S2:** LRP1 plays the beneficial effects for dopaminergic neurons uptake of α‐Syn‐HDO‐1. (A) The representative image of TH and FAM for immunofluorescence staining. Scale bar = 100 μm. (B) The qPCR for α‐Syn in the SNc (mean ± SEM, *n* = 6 per group). (C) The representative image and quantification analysis of α‐Syn in the SNc for western blot assay (mean ± SEM, *n* = 8 per group). (D) The representative image of LRP1 and biotin for immunofluorescence staining. After treatment with or without biotin‐α‐Syn HDO‐1 30 min, neuro‐2a cells were stained with an LRP1–specific antibody (red) for LRP1 and streptavidin‐FITC (green) for biotin, followed by counterstaining with DAPI. Scale bars are = 50 μm. *p < 0.05, **p < 0.01, ***p < 0.001, and ****p < 0.0001 compare with vehicle + α‐Syn‐HDO‐1 group, one‐way ANOVA.
**Figure S3:** LRP1 plays the beneficial effects for dopaminergic neurons uptake of α‐Syn‐HDO‐2. (A) The representative image of TH and CY5 for immunofluorescence staining. Scale bar = 100 μm. (B) The qPCR for α‐Syn in the SNc (mean ± SEM, *n* = 6 per group). (C) The representative image and quantification analysis of α‐Syn in the SNc for western blot assay (mean ± SEM, *n* = 8 per group). (D, E) The representative image of LDLR, LRP1, and Biotin for immunofluorescence staining. After treatment with or without biotin‐α‐Syn‐HDO‐2 30 min, neuro‐2a cells were stained with an LDLR and LRP1–specific antibody (red) for LDLR and LRP1 and streptavidin‐FITC (green) for biotin, followed by counterstaining with DAPI. Scale bars are = 50 μm. *p < 0.05, **p < 0.01, and ***p < 0.001 compare with vehicle + α‐Syn‐HDO‐2 group, one‐way ANOVA.
**Figure S4:** α‐Syn‐HDOs prevent α‐Syn pathology in PFFs‐treated HEK293‐α‐Syn‐YFP cells, primary neurons, and A53T mice. (A, B) The representative image and quantification analysis of aggregated α‐Syn in PFFs‐treated HEK293‐α‐Syn‐YFP cells. Scale bar = 100 μm. (C) The representative image and quantification analysis of p‐α‐Syn and α‐Syn in PFFs‐treated HEK293‐α Syn‐YFP cells for western blot assay (mean ± SEM, *n* = 6 per group). (D) The representative image and quantification analysis of p‐α‐Syn in the PFFs‐treated primary neurons. Scale bar = 50 μm. (E) The representative image and quantification analysis of p‐α‐Syn and α‐Syn in the SNc of A53T mice for western blot assay (mean ± SEM, *n* = 6 per group). **p < 0.01, ***p < 0.001, and ****p < 0.0001 compared with PFFs group, one‐way ANOVA.
**Figure S5:** BDNF inhibits aberrant α‐Syn aggregation in vitro. (A) The representative image and quantification analysis of aggregated α‐Syn in PFFs treated HEK293‐α‐Syn‐YFP cells transfected with TrkB. Scale bar = 100 μm (mean ± SEM, *n* = 5 per group). (B) The representative image and quantification analysis of p‐α Syn, α‐Syn, and the ratio of p‐TrkB/TrkB in PFFs‐treated HEK293‐α‐Syn‐YFP cells transfected with TrkB for western blot assay (mean ± SEM, *n* = 6 per group). *p < 0.05, **p < 0.01, ***p < 0.001, and ****p < 0.0001 compared with PFFs group, one‐way ANOVA.
**Figure S6:** 7,8‐DHF inhibits aberrant α‐Syn aggregation in vitro. (A) The representative image and quantification analysis of aggregated α‐Syn in PFFs treated HEK293‐α‐Syn‐YFP cells transfected with TrkB. Scale bar = 100 μm (mean ± SEM, *n* = 6 per group). (B) The representative image and quantification analysis of p‐α Syn, α‐Syn, and the ratio of p‐TrkB/TrkB in PFFs‐treated HEK293‐α‐Syn‐YFP cells transfected with TrkB for western blot assay (mean ± SEM, *n* = 6 per group). *p < 0.05, **p < 0.01, ***p < 0.001, and ****p < 0.0001 compared with PFFs group, one‐way ANOVA.
**Figure S7:** The p‐α‐Syn and BDNF levels in the SNc. (A–F) The p‐α‐Syn and BDNF levels in the SNc for ELSIA assay (mean ± SEM, *n* = 6 per group). *p < 0.05, **p < 0.01, and ***p < 0.001 compared with PFFs group or α Syn‐HDO‐1‐ or α‐Syn‐HDO‐2‐treated mice following PFFs administration, one‐way ANOVA.

## Data Availability

Additional information necessary for reanalysis of the data presented in this paper can be obtained from the lead contact upon request.
