# Supplementary material for: Inhibition of Abnormal Elevated α‐Synuclein Ameliorates Dopaminergic Neuron Degeneration in Parkinson's Disease Mouse Model
Source: Aging Cell. 2026 Sep 23;25(10):e70717. doi: 10.1111/acel.70717 (PMC13599363; doi:10.1111/acel.70717)
Supplement: Supplementary file 1 [file ACEL-25-e70717-s001.pdf]

## Supplement figure

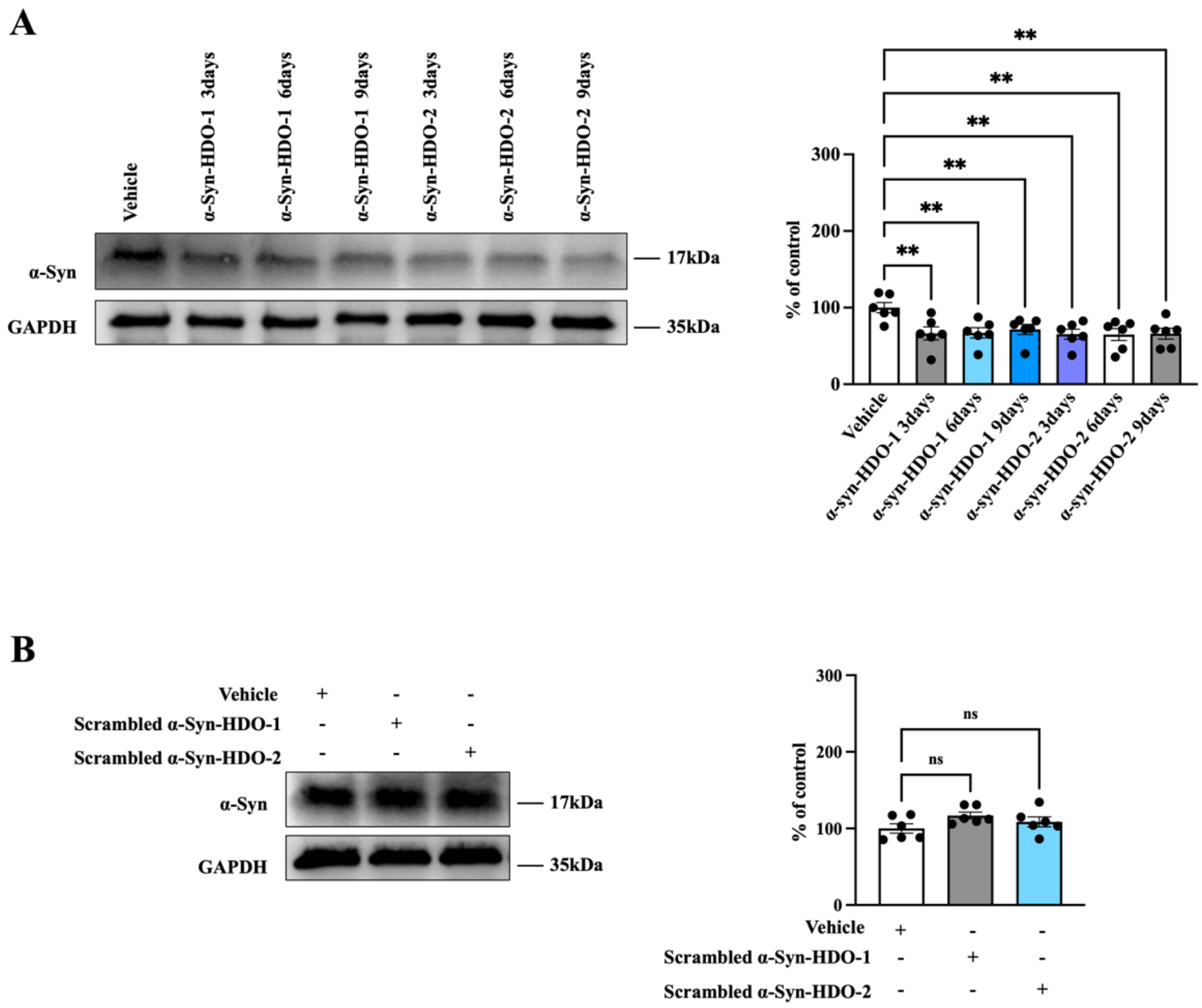


**Supplement figure 1: α-Syn-HDOs exhibit a long-term silencing effect on α-Syn**

**A**: Representative western blot images and quantification of α-Syn levels in the SNc following vehicle or α-Syn-HDOs injection. (Mean ± SEM, n = 6 per group). **B**: Representative western blot images and quantification of α-Syn levels in the SNc following vehicle or scrambled α-Syn-HDOs injection. (Mean ± SEM, n = 6 per group). *p < 0.05 and **p < 0.01 compared with vehicle group, one-way ANOVA. ns, not significant.

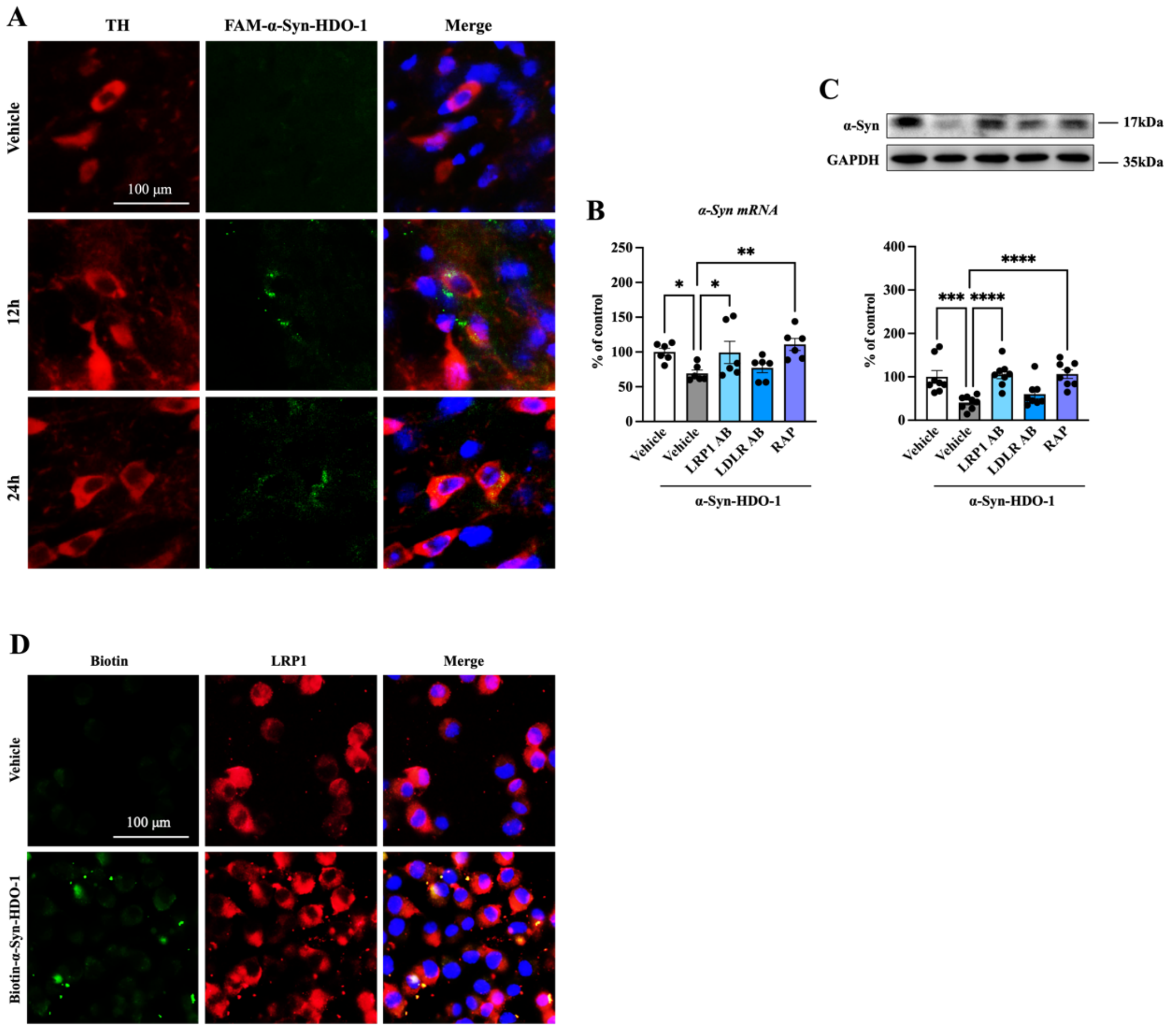


**Supplement figure 2: LRP1 plays the beneficial effects for dopaminergic neurons uptake of α-Syn-HDO-1**

**A**: The representative image of TH and FAM for immunofluorescence staining. Scale bar = 100 μm. **B**: The qPCR for α-Syn in the SNc. (Mean ± SEM, n = 6 per group). **C**: The representative image and quantification analysis of α-Syn in the SNc for western blot assay. (Mean ± SEM, n = 8 per group). **D**: The representative image of LRP1 and Biotin for immunofluorescence staining. After treatment with or without biotin-α-Syn-HDO-1 30 minutes, neuro-2a cells were stained with an LRP1–specific antibody (red) for LRP1 and streptavidin-FITC (green) for biotin, followed by counterstaining with DAPI. Scale bars are = 50 μm. *$p < 0.05$, **$p < 0.01$, ***$p < 0.001$, and ****$p < 0.0001$ compare with vehicle + α-Syn-HDO-1 group, one-way ANOVA.

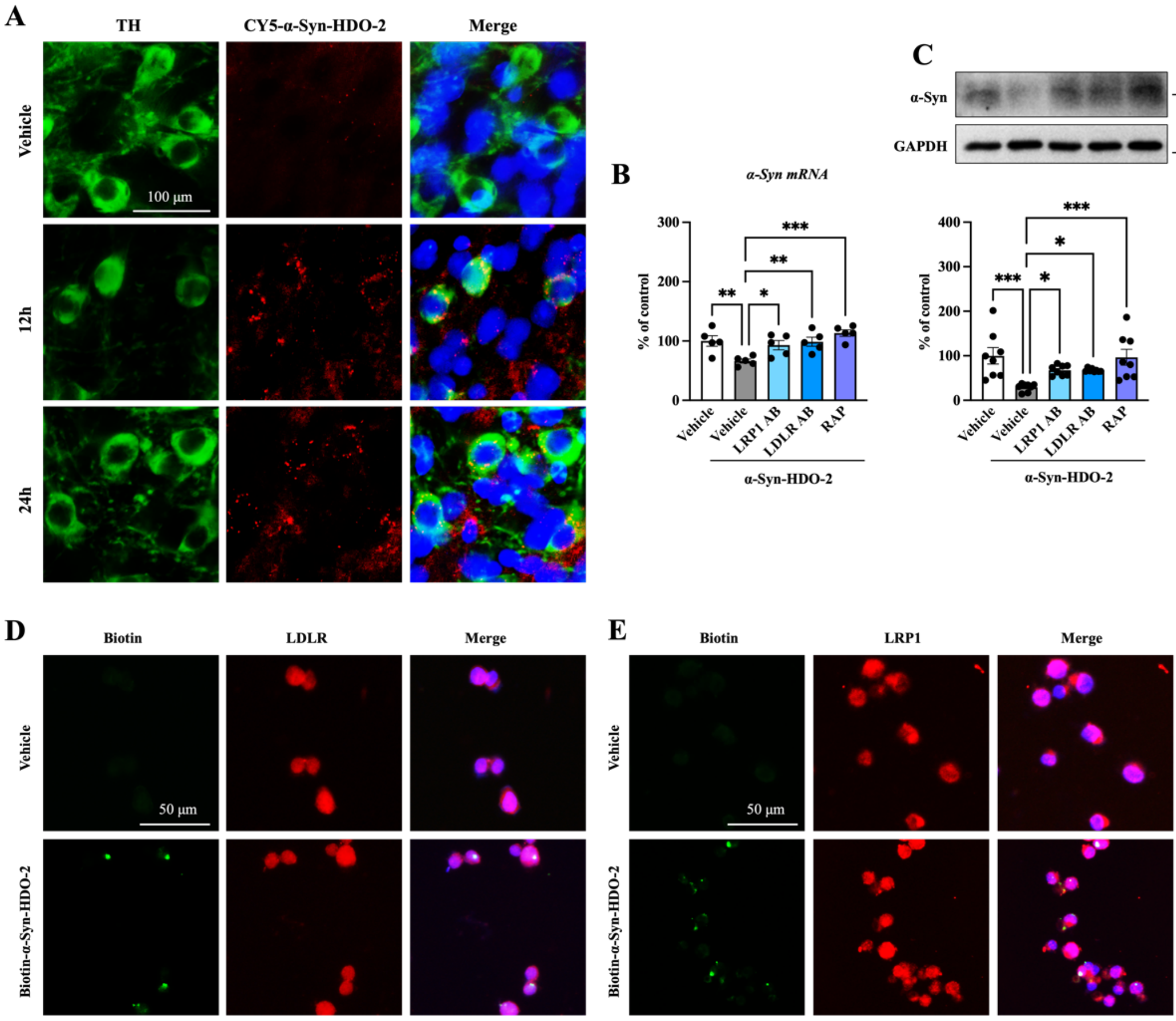


**Supplement figure 3: LRP1 plays the beneficial effects for dopaminergic neurons uptake of α-Syn-HDO-2**

**A**: The representative image of TH and CY5 for immunofluorescence staining. Scale bar = 100 μm. **B**: The qPCR for α-Syn in the SNc. (Mean ± SEM, n = 6 per group). **C**: The representative image and quantification analysis of α-Syn in the SNc for western blot assay. (Mean ± SEM, n = 8 per group). **D and E**: The representative image of LDLR, LRP1, and Biotin for immunofluorescence staining. After treatment with or without biotin-α-Syn-HDO-2 30 minutes, neuro-2a cells were stained with an LDLR and LRP1–specific antibody (red) for LDLR and LRP1 and streptavidin-FITC (green) for biotin, followed by counterstaining with DAPI. Scale bars are = 50 μm. *$p < 0.05$, **$p < 0.01$, and ***$p < 0.001$ compare with vehicle + α-Syn-HDO-2 group, one-way ANOVA.

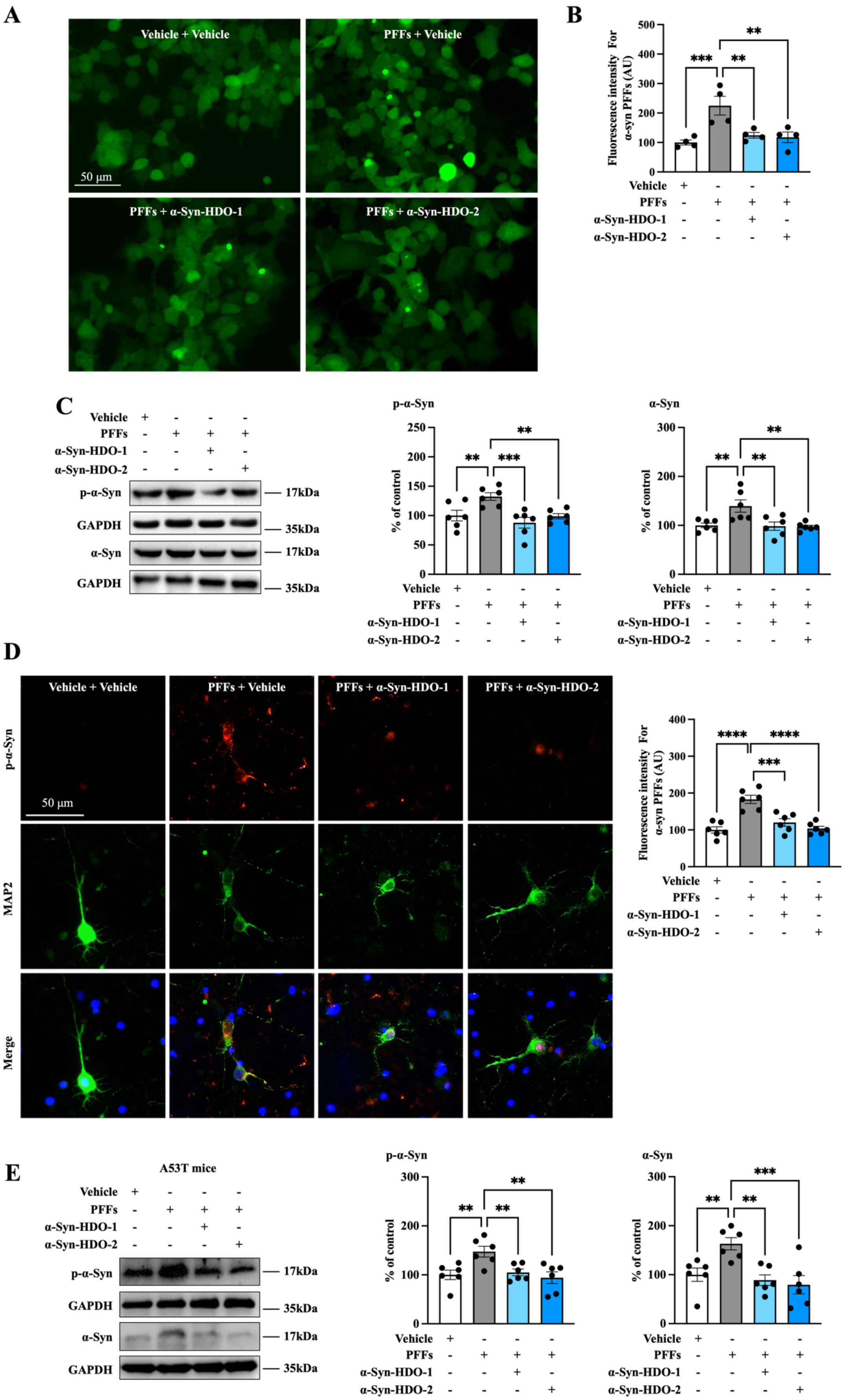

A
Vehicle + Vehicle
PFFs + Vehicle
PFFs + α-Syn-HDO-1
PFFs + α-Syn-HDO-2
50 μm
B
Fluorescence intensity For α-syn PFFs (AU)
Vehicle
PFFs
α-Syn-HDO-1
α-Syn-HDO-2
C
Vehicle
PFFs
α-Syn-HDO-1
α-Syn-HDO-2
p-α-Syn
GAPDH
α-Syn
GAPDH
17kDa
35kDa
17kDa
35kDa
p-α-Syn
α-Syn
% of control
D
Vehicle + Vehicle
PFFs + Vehicle
PFFs + α-Syn-HDO-1
PFFs + α-Syn-HDO-2
p-α-Syn
MAP2
Merge
50 μm
Fluorescence intensity For α-syn PFFs (AU)
E
A53T mice
Vehicle
PFFs
α-Syn-HDO-1
α-Syn-HDO-2
p-α-Syn
GAPDH
α-Syn
GAPDH
17kDa
35kDa
17kDa
35kDa
p-α-Syn
α-Syn
% of control

**Supplement figure 4. α-Syn-HDOs prevent α-Syn pathology in PFFs-treated HEK293-α-Syn-YFP cells, primary neurons, and A53T mice**

**A and B**: The representative image and quantification analysis of aggregated α-Syn in PFFs-treated HEK293-α-Syn-YFP cells. Scale bar = 100 μm. **C:** The representative image and quantification analysis of p-α-Syn and α-Syn in PFFs-treated HEK293-α-Syn-YFP cells for western blot assay. (Mean ± SEM, n = 6 per group). **D**: The representative image and quantification analysis of p-α-Syn in the PFFs-treated primary neurons. Scale bar = 50 μm. **E**: The representative image and quantification analysis of p-α-Syn and α-Syn in the SNc of A53T mice for western blot assay. (Mean ± SEM, n = 6 per group). $^{**}p < 0.01$, $^{***}p < 0.001$, and $^{****}p < 0.0001$ compared with PFFs group, one-way ANOVA.

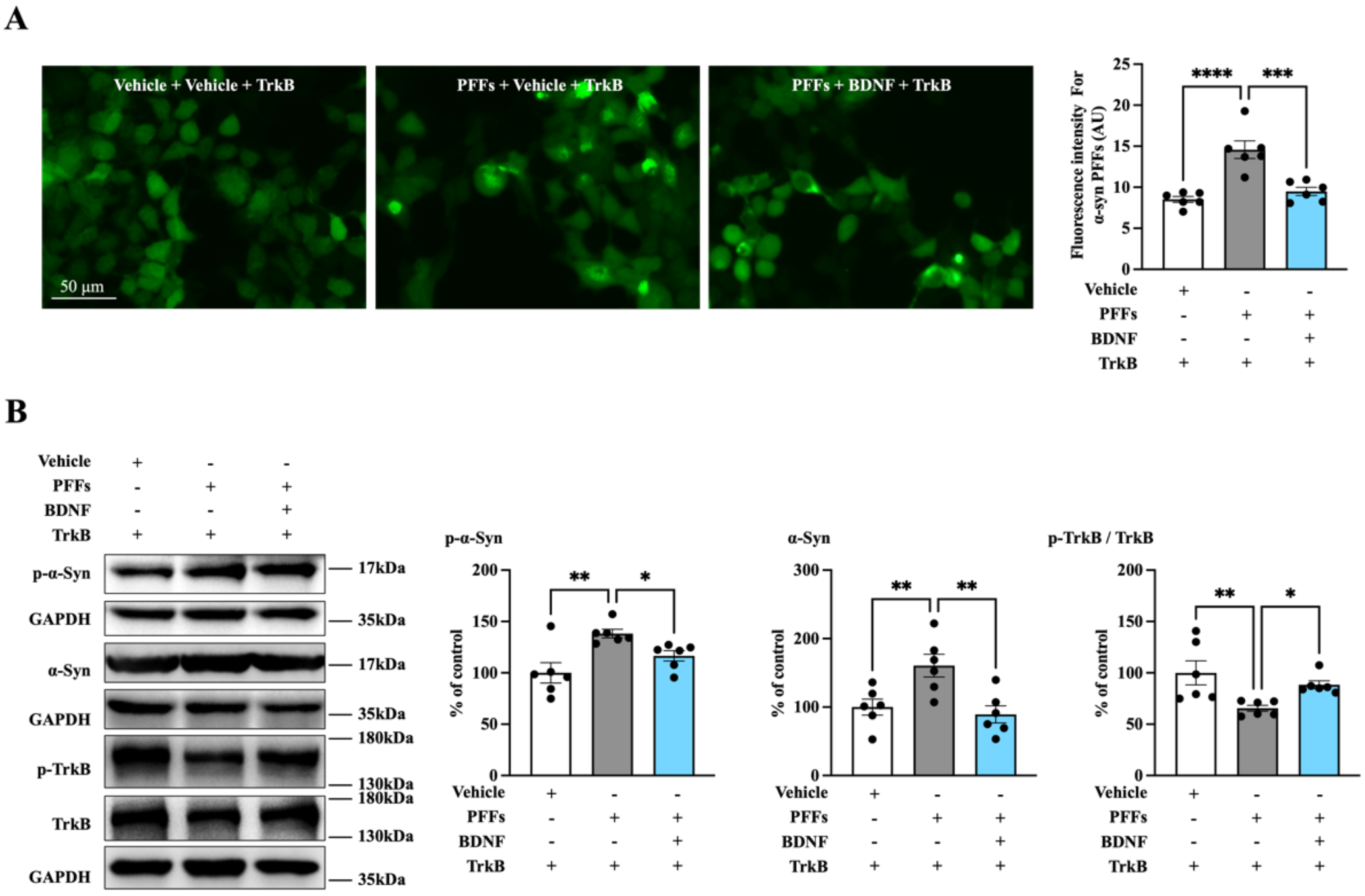


**Supplement figure 5. BDNF inhibits aberrant α-Syn aggregation *in vitro***

**A**: The representative image and quantification analysis of aggregated α-Syn in PFFs-treated HEK293-α-Syn-YFP cells transfected with TrkB. Scale bar = 100 µm. (Mean ± SEM, n = 5 per group). **B**: The representative image and quantification analysis of p-α-Syn, α-Syn, and the ratio of p-TrkB/TrkB in PFFs-treated HEK293-α-Syn-YFP cells transfected with TrkB for western blot assay. (Mean ± SEM, n = 6 per group). *$p < 0.05$, **$p < 0.01$, ***$p < 0.001$, and ****$p < 0.0001$ compared with PFFs group, one-way ANOVA.

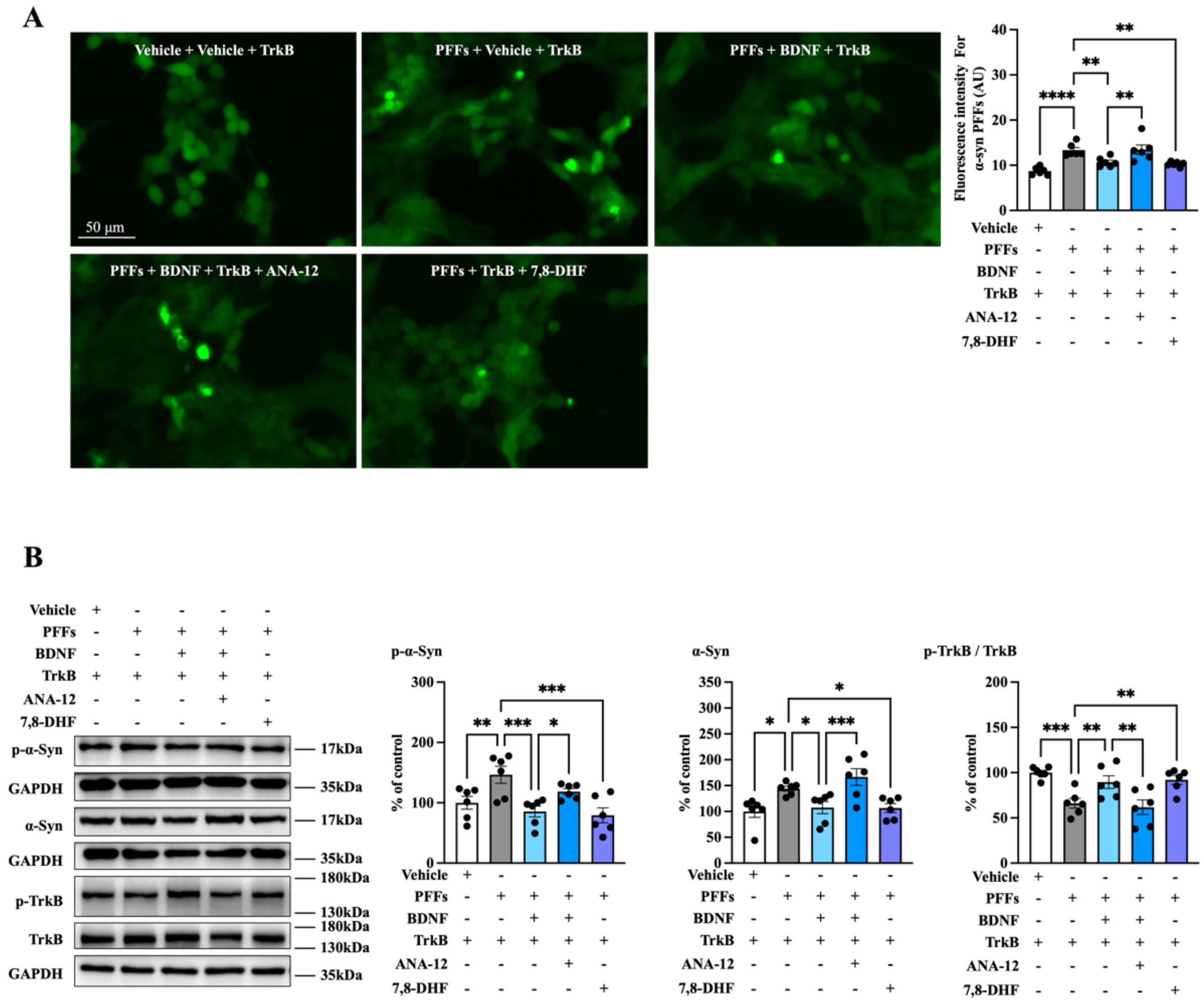


**Supplement figure 6. 7,8-DHF inhibits aberrant α-Syn aggregation *in vitro***

**A**: The representative image and quantification analysis of aggregated α-Syn in PFFs-treated HEK293-α-Syn-YFP cells transfected with TrkB. Scale bar = 100 μm. (Mean ± SEM, n = 6 per group). **B**: The representative image and quantification analysis of p-α-Syn, α-Syn, and the ratio of p-TrkB/TrkB in PFFs-treated HEK293-α-Syn-YFP cells transfected with TrkB for western blot assay. (Mean ± SEM, n = 6 per group). $*p < 0.05$, $**p < 0.01$, $***p < 0.001$, and $****p < 0.0001$ compared with PFFs group, one-way ANOVA.

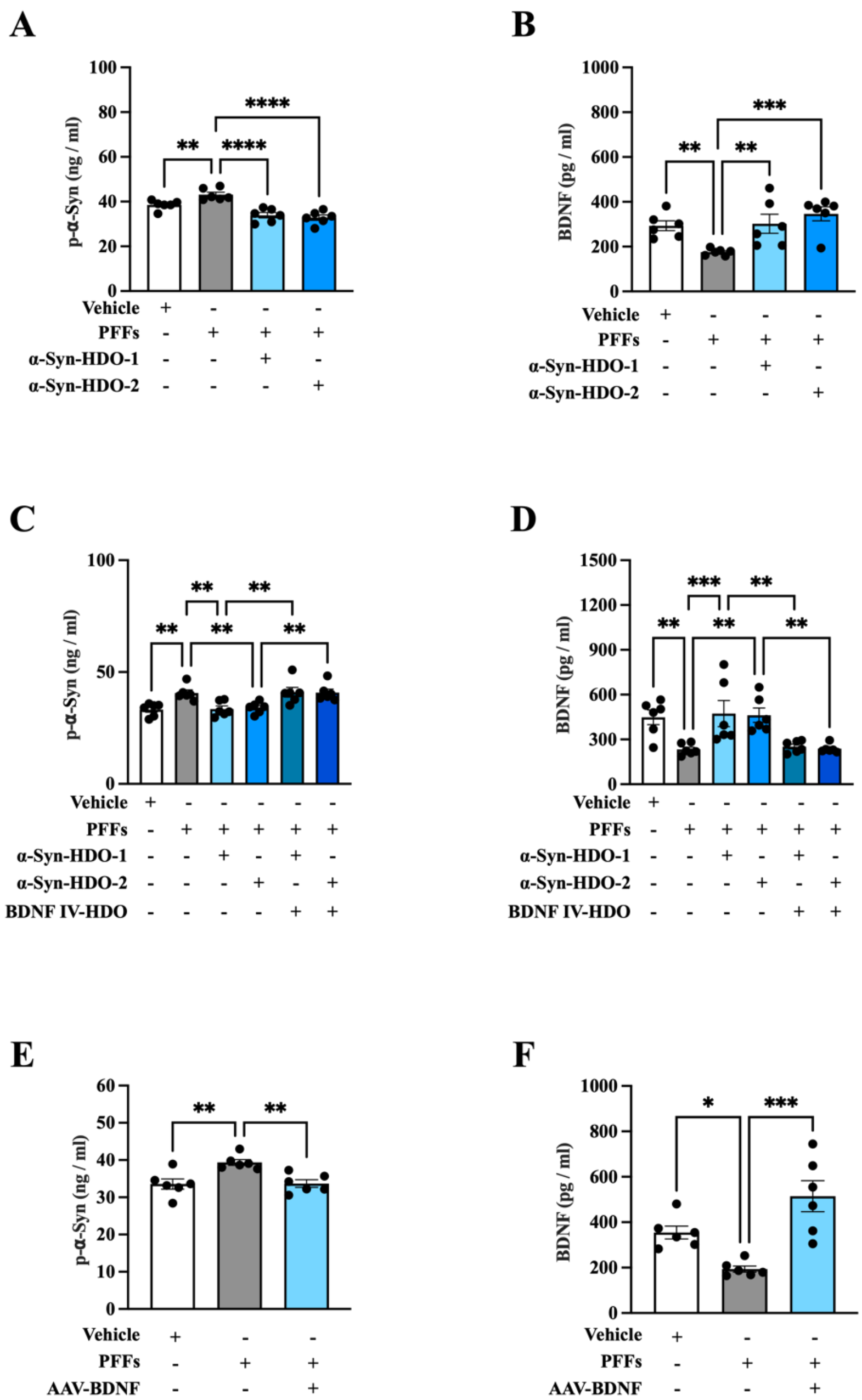


**Supplement figure 7. The p-α-Syn and BDNF levels in the SNc**

**A - F**: The p-α-Syn and BDNF levels in the SNc for ELSIA assay. (Mean ± SEM, n = 6 per group). *p < 0.05, **p < 0.01, and ***p < 0.001 compared with PFFs group or α-Syn-HDO-1- or α-Syn-HDO-2-treated mice following PFFs administration, one-way ANOVA.
